# Supplementary material for: Higher-order phosphatase–substrate contacts terminate the integrated stress response
Source: Nat Struct Mol Biol. 2021 Oct 8;28(10):835–46. doi: 10.1038/s41594-021-00666-7 (PMC8500838; doi:10.1038/s41594-021-00666-7)
Supplement: Supplementary file 1 — Description of chemicals, cell lines and plasmids used in this study and validation reports for new structures. [file 41594_2021_666_MOESM1_ESM.pdf]

nature portfolio

https://doi.org/10.1038/s41594-021-00666-7

Supplementary information

Higher-order phosphatase–substrate contacts terminate the integrated stress response

In the format provided by the authors and unedited

Supplementary Table 1

REAGENT or RESOURCE	SOURCE	IDENTIFIER
**Bacterial and virus strains**		
T7 Express lysY/Iq	New England Biolabs	Cat. No. C3013
CHO-K1 with CHOP::GFP reporter	PMID:11381086	C30
**Chemicals, peptides, and recombinant proteins**		
Thapsigargin	Calbiochem	586005-1MG
Sodium arsenite	Sigma	S7400
PhosTag reagent	Generon	F4002-10mg
Latrunculin B	AbCam	#ab14491
Partially purified (DP grade) bovine pancreatic DNase I	Worthington	#LS002139
Lipofectamine LTX with Plus reagent	ThermoFisher	A12621
Streptavidin-coated BLI Probes	Fortebio	18-5020
**Recombinant DNA (first mentioned in the indicated figure)**		
UK2514_Smt3_huPPP1R15A_582-621_pSUMO3 (Fig 1b)	here	UK2514
UK2699_hR15A_553-624_MBP_rPP1A_7-300_pSUMO3 (Fig 2a)	here	UK2699
UK2750_hR15A_553-624-R595E-MBP_rPP1A_7-300_pSUMO3 (Fig 2a)	here	UK2750
UK2751_hR15A_553-624-F592A_R595A-MBP_rPP1A_7-300_pSUMO3 (Fig 2a)	here	UK2751
UK2752_hR15A_553-624-R612A-MBP_rPP1A_7-300_pSUMO3 (Fig 2)	here	UK2752
UK2755_hR15A_553-624-F592A_R595E-MBP_rPP1A_7-300_pSUMO3 (Fig 2a)	here	UK2755
UK2731_heIF2a_2-187_WT_pSUMO3 (Fig 2a)	(Zyryanova et al., 2021)	UK2731
UK168_PerkKD-pGEX4T-1 (Fig 2a)	(Harding et al., 1999)	UK168

UK2773_hR15A_583-621_WT_AviTag_H6_pET-30a(+) (Fig 2d)		UK2773
UK2776_hR15A_583-621_R595E_AviTag_H6_pET-30a(+)(Fig 2d)		UK2776
UK2779_hR15A_583-621_F592A_R595E_AviTag_H6_pET-30a(+)(Fig 2d)		UK2779
UK2777_hR15A_583-621_F592A_R595A_AviTag_H6_pET-30a(+)(Fig 2d)		UK2777
UK1250_FLAG_mR15A_1-653_mCherry (Fig 2)	(Chen et al., 2015)	UK1250
UK1302_FLAG_mR15A_1-653_F585A_mCherry (Fig 2)	(Chen et al., 2015)	UK1302
UK2724_FLAG_mR15A_1-653_F585A_R588E_mCherry (Fig 2e)	here	UK2724
UK2727_FLAG_mR15A_1-653_R588E_mCherry (Fig 2e)	here	UK2727
UK2775_FLAG_mR15A_1-653_F585A_R588A_mCherry (Fig 2e)	here	UK2775
UK2788_FLAG_mR15A_1-653_R605A_mCherry (Fig 2e)	here	UK2788
UK2830_p-eGFP-G3BP (Fig 2h)	(Panas et al., 2019)	UK2830
UK1300_FLAG_mR15A_1-653_W575A_mCherry (Fig 3)	(Chen et al., 2015)	UK1300
UK2739_pCEFL_BFP (Fig 3)	Here	UK2739
UK2740_hbActin_2-376_A204E_P243K_pCEFL_BFP (Fig 3)	here	UK2740
UK2741_hbActin_2-376_A204E_P243K_E336R_pCEFL_BFP (Fig 3)	here	UK2741
UK2720_hR15A_553-624-MBP_rPP1A_7-300_D64A_pSUMO3 (Fig 4)	here	UK2720
UK2814_hR15A_553-624_R591A_MBP_rPP1A_7-300_pSUMO3 (Fig 5a)	here	UK2814
UK2815_hR15A_553-624_R594A_MBP_rPP1A_7-300_pSUMO3 (Fig 5a)	here	UK2815
UK2826_hR15A_553-624_W582A_MBP_rPP1A_7-300_pSUMO3 (Fig 5a)	here	UK2826

UK2827_hR15A_553-624_R591A_R594A_MBP_rPP1A_7-300_pSUMO3 (Fig 5a)	here	UK2827
UK2808_FLAG_mR15A_1-653_R584A_mCherry (Fig 5d)	here	UK2808
UK2809_FLAG_mR15A_1-653_R587A_mCherry (Fig 5d)	here	UK2809
UK2733_heIF2a_2-187_WT_AviTag_H6_pET-30a(+) (Fig 5f)	(Zyryanova et al., 2021)	UK2733
UK2840_hR15A_553-624_W582A_MBP_rPP1A_7-300_D64A_pSUMO3 (Fig 5f)	here	UK2840
UK2841_hR15A_553-624_R591A_MBP_rPP1A_7-300_D64A_pSUMO3 (Fig 5f)	here	UK2841
UK2849_heIF2a_2-187_M44A_pSUMO3 (Fig 6b and c)	here	UK2849
UK2850_heIF2a_2-187_K79A_pSUMO3 (Fig 6b and c)	here	UK2850
UK2851_heIF2a_2-187_Y81A_pSUMO3 (Fig 6b and c)	here	UK2851
UK2852_heIF2a_2-187_D83A_pSUMO3 (Fig 6c)	here	UK2852

Full wwPDB X-ray Structure Validation Report (i)

Mar 28, 2021 – 12:47 am GMT

PDB ID	:	7NXV
Title	:	Crystal structure of the complex of DNase I/G-actin/PPP1R15A_582-621
Deposited on	:	2021-03-19
Resolution	:	2.55 Å(reported)

This is a Full wwPDB X-ray Structure Validation Report.

This report is produced by the wwPDB biocuration pipeline after annotation of the structure.

We welcome your comments at *validation@mail.wwpdb.org*
A user guide is available at
https://www.wwpdb.org/validation/2017/XrayValidationReportHelp
with specific help available everywhere you see the (i) symbol.

CONFIDENTIAL VALIDATION REPORT

The following versions of software and data (see references (i)) were used in the production of this report:

MolProbity	:	4.02b-467
Mogul	:	1.8.5 (274361), CSD as541be (2020)
Xtriage (Phenix)	:	1.13
EDS	:	2.18
buster-report	:	1.1.7 (2018)
Percentile statistics	:	20191225.v01 (using entries in the PDB archive December 25th 2019)
Refmac	:	5.8.0158
CCP4	:	7.0.044 (Gargrove)
Ideal geometry (proteins)	:	Engh & Huber (2001)
Ideal geometry (DNA, RNA)	:	Parkinson et al. (1996)
Validation Pipeline (wwPDB-VP)	:	2.18

1 Overall quality at a glance (i)

The following experimental techniques were used to determine the structure:
X-RAY DIFFRACTION

The reported resolution of this entry is 2.55 Å.

Percentile scores (ranging between 0-100) for global validation metrics of the entry are shown in the following graphic. The table shows the number of entries on which the scores are based.

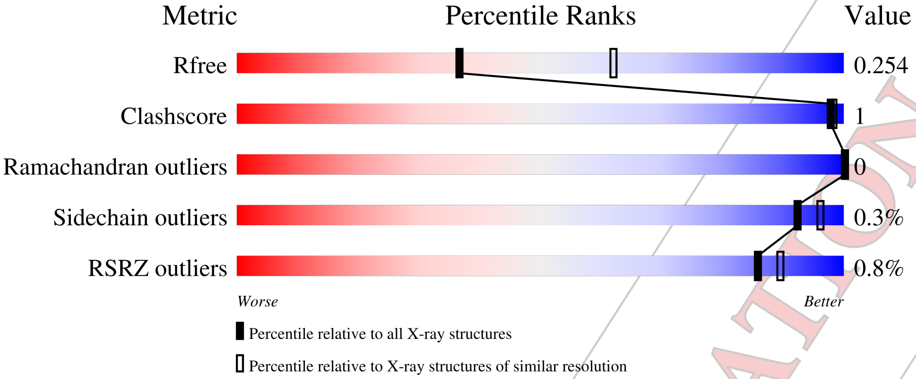

Metric	Whole archive (#Entries)	Similar resolution (#Entries, resolution range(Å))
R_{free}	130704	1284 (2.56-2.52)
Clashscore	141614	1332 (2.56-2.52)
Ramachandran outliers	138981	1315 (2.56-2.52)
Sidechain outliers	138945	1315 (2.56-2.52)
RSRZ outliers	127900	1272 (2.56-2.52)

The table below summarises the geometric issues observed across the polymeric chains and their fit to the electron density. The red, orange, yellow and green segments of the lower bar indicate the fraction of residues that contain outliers for >=3, 2, 1 and 0 types of geometric quality criteria respectively. A grey segment represents the fraction of residues that are not modelled. The numeric value for each fraction is indicated below the corresponding segment, with a dot representing fractions <=5% The upper red bar (where present) indicates the fraction of residues that have poor fit to the electron density. The numeric value is given above the bar.

Mol	Chain	Length	Quality of chain
1	A	375	% 98% ••
1	D	375	% 97% ••
2	B	260	97% •
2	F	260	99% •
3	C	40	5% 88% 8% 5%

Continued on next page...

CONFIDENTIAL VALIDATION REPORT

Continued from previous page...

Mol	Chain	Length	Quality of chain
3	E	40	5% / 92% / 5% / •
4	G	2	100%
4	H	2	50% / 50%

CONFIDENTIAL VALIDATION REPORT

2 Entry composition (i)

There are 7 unique types of molecules in this entry. The entry contains 10650 atoms, of which 0 are hydrogens and 0 are deuteriums.

In the tables below, the ZeroOcc column contains the number of atoms modelled with zero occupancy, the AltConf column contains the number of residues with at least one atom in alternate conformation and the Trace column contains the number of residues modelled with at most 2 atoms.

- Molecule 1 is a protein called Actin, alpha skeletal muscle, intermediate form.

Mol	Chain	Residues	Atoms					ZeroOcc	AltConf	Trace
1	A	373	Total 2908	C 1840	N 490	O 557	S 21	0	0	0
1	D	371	Total 2895	C 1833	N 488	O 553	S 21	0	0	0

- Molecule 2 is a protein called Deoxyribonuclease-1.

Mol	Chain	Residues	Atoms					ZeroOcc	AltConf	Trace
2	B	260	Total 2049	C 1298	N 341	O 402	S 8	0	0	0
2	F	260	Total 2049	C 1298	N 341	O 402	S 8	0	0	0

- Molecule 3 is a protein called Protein phosphatase 1 regulatory subunit 15A.

Mol	Chain	Residues	Atoms					ZeroOcc	AltConf	Trace
3	C	38	Total 280	C 173	N 55	O 51	S 1	0	0	0
3	E	39	Total 295	C 180	N 61	O 53	S 1	0	0	0

- Molecule 4 is an oligosaccharide called 2-acetamido-2-deoxy-beta-D-glucopyranose-(1-4)-2-acetamido-2-deoxy-beta-D-glucopyranose.

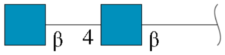

Mol	Chain	Residues	Atoms				ZeroOcc	AltConf	Trace
4	G	2	Total 28	C 16	N 2	O 10	0	0	0

Continued on next page...

CONFIDENTIAL VALIDATION REPORT

Continued from previous page...

Mol	Chain	Residues	Atoms	ZeroOcc	AltConf	Trace
4	H	2	Total C N O<br>28 16 2 10	0	0	0

- Molecule 5 is ADENOSINE-5'-TRIPHOSPHATE (three-letter code: ATP) (formula: $C_{10}H_{16}N_5O_{13}P_3$).

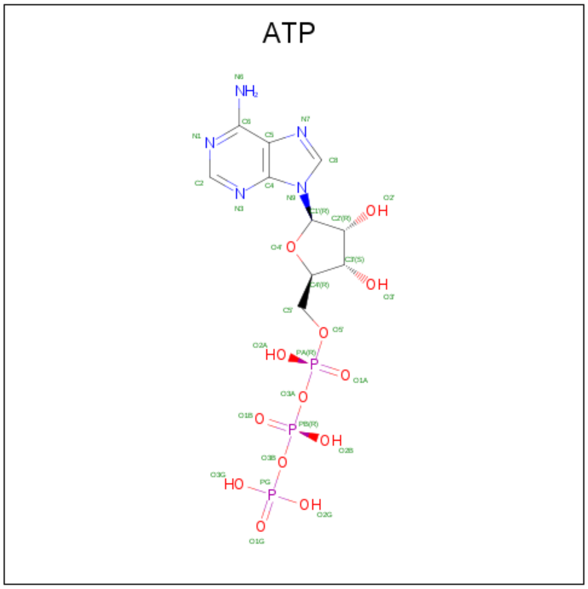

Mol	Chain	Residues	Atoms	ZeroOcc	AltConf
5	A	1	Total C N O P<br>31 10 5 13 3	0	0
5	D	1	Total C N O P<br>31 10 5 13 3	0	0

- Molecule 6 is CALCIUM ION (three-letter code: CA) (formula: Ca).

Mol	Chain	Residues	Atoms	ZeroOcc	AltConf
6	A	1	Total Ca<br>1 1	0	0
6	B	2	Total Ca<br>2 2	0	0
6	D	1	Total Ca<br>1 1	0	0
6	F	2	Total Ca<br>2 2	0	0

- Molecule 7 is water.

CONFIDENTIAL VALIDATION REPORT

Mol	Chain	Residues	Atoms	ZeroOcc	AltConf
7	A	13	Total O<br>13 13	0	0
7	B	8	Total O<br>8 8	0	0
7	D	11	Total O<br>11 11	0	0
7	E	1	Total O<br>1 1	0	0
7	F	17	Total O<br>17 17	0	0

CONFIDENTIAL VALIDATION REPORT

3 Residue-property plots (i)

These plots are drawn for all protein, RNA, DNA and oligosaccharide chains in the entry. The first graphic for a chain summarises the proportions of the various outlier classes displayed in the second graphic. The second graphic shows the sequence view annotated by issues in geometry and electron density. Residues are color-coded according to the number of geometric quality criteria for which they contain at least one outlier: green = 0, yellow = 1, orange = 2 and red = 3 or more. A red dot above a residue indicates a poor fit to the electron density (RSRZ > 2). Stretches of 2 or more consecutive residues without any outlier are shown as a green connector. Residues present in the sample, but not in the model, are shown in grey.

- Molecule 1: Actin, alpha skeletal muscle, intermediate form

Chain A:
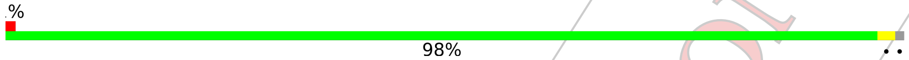

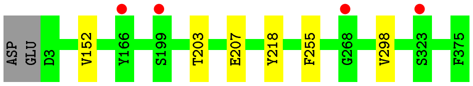

- Molecule 1: Actin, alpha skeletal muscle, intermediate form

Chain D:
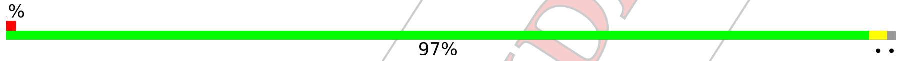

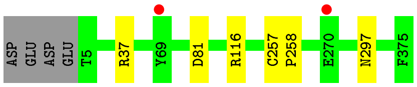

- Molecule 2: Deoxyribonuclease-1

Chain B:
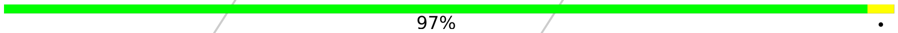

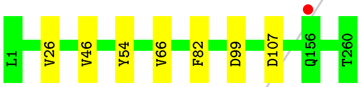

- Molecule 2: Deoxyribonuclease-1

Chain F:
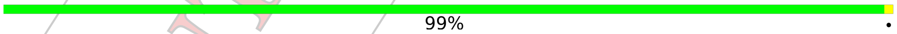

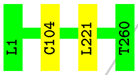

- Molecule 3: Protein phosphatase 1 regulatory subunit 15A

Chain C:
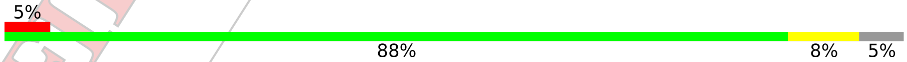

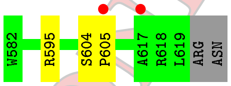

- Molecule 3: Protein phosphatase 1 regulatory subunit 15A

CONFIDENTIAL VALIDATION REPORT

Chain E:

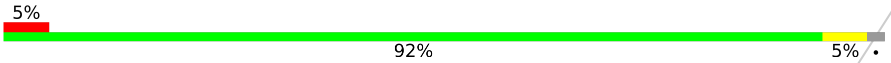

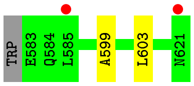

• Molecule 4: 2-acetamido-2-deoxy-beta-D-glucopyranose-(1-4)-2-acetamido-2-deoxy-beta-D-gluc opyranose

Chain G:

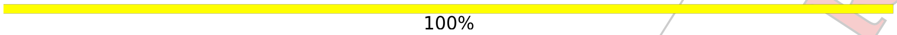

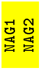

• Molecule 4: 2-acetamido-2-deoxy-beta-D-glucopyranose-(1-4)-2-acetamido-2-deoxy-beta-D-gluc opyranose

Chain H:

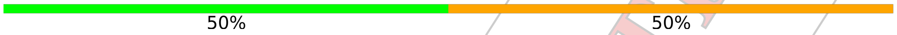

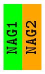

CONFIDENTIAL VALIDATION REPORT

4 Data and refinement statistics (i)

Property	Value	Source
Space group	P 21 21 21	Depositor
Cell constants<br>a, b, c, α, β, γ	86.52Å 107.81Å 192.41Å<br>90.00° 90.00° 90.00°	Depositor
Resolution (Å)	55.11 – 2.55<br>55.12 – 2.55	Depositor<br>EDS
% Data completeness<br>(in resolution range)	99.8 (55.11-2.55)<br>99.5 (55.12-2.55)	Depositor<br>EDS
R_{merge}	0.32	Depositor
R_{sym}	(Not available)	Depositor
$< I/\sigma(I) >$ [1]	1.72 (at 2.55Å)	Xtriage
Refinement program	REFMAC 5.8.0267	Depositor
R, R_{free}	0.216 , 0.248<br>0.223 , 0.254	Depositor<br>DCC
R_{free} test set	2892 reflections (4.89%)	wwPDB-VP
Wilson B-factor (Å^2)	34.8	Xtriage
Anisotropy	0.185	Xtriage
Bulk solvent k_{sol}(e/Å^3), B_{sol}(Å^2)	0.35 , 29.7	EDS
L-test for twinning[2]	$< \|L\| > = 0.50$, $< L^2 > = 0.33$	Xtriage
Estimated twinning fraction	No twinning to report.	Xtriage
F_o,F_c correlation	0.92	EDS
Total number of atoms	10650	wwPDB-VP
Average B, all atoms (Å^2)	39.0	wwPDB-VP

Xtriage's analysis on translational NCS is as follows: *The largest off-origin peak in the Patterson function is 2.93% of the height of the origin peak. No significant pseudotranslation is detected.*

[1] Intensities estimated from amplitudes.

[2] Theoretical values of $< |L| >$, $< L^2 >$ for acentric reflections are 0.5, 0.333 respectively for untwinned datasets, and 0.375, 0.2 for perfectly twinned datasets.

CONFIDENTIAL VALIDATION REPORT

5 Model quality (i)

5.1 Standard geometry (i)

Bond lengths and bond angles in the following residue types are not validated in this section: CA, NAG, ATP

The Z score for a bond length (or angle) is the number of standard deviations the observed value is removed from the expected value. A bond length (or angle) with $|Z| > 5$ is considered an outlier worth inspection. RMSZ is the root-mean-square of all Z scores of the bond lengths (or angles).

Mol	Chain	Bond lengths		Bond angles	
		RMSZ	$\#\|Z\|>5$	RMSZ	$\#\|Z\|>5$
1	A	0.65	0/2971	0.71	0/4026
1	D	0.65	0/2958	0.70	0/4008
2	B	0.65	0/2095	0.72	0/2853
2	F	0.65	0/2095	0.72	0/2853
3	C	0.67	0/284	0.74	0/386
3	E	0.67	0/299	0.72	0/404
All	All	0.65	0/10702	0.71	0/14530

There are no bond length outliers.

There are no bond angle outliers.

There are no chirality outliers.

There are no planarity outliers.

5.2 Too-close contacts (i)

In the following table, the Non-H and H(model) columns list the number of non-hydrogen atoms and hydrogen atoms in the chain respectively. The H(added) column lists the number of hydrogen atoms added and optimized by MolProbity. The Clashes column lists the number of clashes within the asymmetric unit, whereas Symm-Clashes lists symmetry-related clashes.

Mol	Chain	Non-H	H(model)	H(added)	Clashes	Symm-Clashes
1	A	2908	0	2866	4	0
1	D	2895	0	2860	2	0
2	B	2049	0	1980	4	0
2	F	2049	0	1981	1	0
3	C	280	0	263	2	0
3	E	295	0	282	1	0
4	G	28	0	25	0	0

Continued on next page...

CONFIDENTIAL VALIDATION REPORT

Continued from previous page...

Mol	Chain	Non-H	H(model)	H(added)	Clashes	Symm-Clashes
4	H	28	0	25	2	0
5	A	31	0	12	0	0
5	D	31	0	12	0	0
6	A	1	0	0	0	0
6	B	2	0	0	0	0
6	D	1	0	0	0	0
6	F	2	0	0	0	0
7	A	13	0	0	0	0
7	B	8	0	0	0	0
7	D	11	0	0	0	0
7	E	1	0	0	0	0
7	F	17	0	0	0	0
All	All	10650	0	10306	14	0

The all-atom clashscore is defined as the number of clashes found per 1000 atoms (including hydrogen atoms). The all-atom clashscore for this structure is 1.

All (14) close contacts within the same asymmetric unit are listed below, sorted by their clash magnitude.

Atom-1	Atom-2	Interatomic distance (Å)	Clash overlap (Å)
4:H:2:NAG:H3	4:H:2:NAG:H83	1.83	0.58
1:A:218:TYR:O	1:A:255:PHE:HA	2.12	0.49
1:D:37:ARG:NH1	1:D:81:ASP:OD1	2.46	0.48
2:B:26:VAL:HG21	2:B:54:TYR:CD1	2.49	0.47
1:A:203:THR:HG21	2:B:46:VAL:HG21	1.96	0.47
1:A:203:THR:O	1:A:207:GLU:HG2	2.15	0.46
2:B:66:VAL:HB	2:B:82:PHE:HB2	2.01	0.43
1:D:257:CYS:HB3	1:D:258:PRO:HD3	2.02	0.42
4:H:2:NAG:H3	4:H:2:NAG:C8	2.49	0.42
2:B:99:ASP:O	2:B:107:ASP:HA	2.20	0.41
3:E:599:ALA:O	3:E:603:LEU:HB2	2.19	0.41
3:C:604:SER:N	3:C:605:PRO:CD	2.83	0.41
3:C:595:ARG:CZ	2:F:104:CYS:SG	3.09	0.41
1:A:152:VAL:HA	1:A:298:VAL:O	2.21	0.40

There are no symmetry-related clashes.

CONFIDENTIAL VALIDATION REPORT

5.3 Torsion angles (i)

5.3.1 Protein backbone (i)

In the following table, the Percentiles column shows the percent Ramachandran outliers of the chain as a percentile score with respect to all X-ray entries followed by that with respect to entries of similar resolution.

The Analysed column shows the number of residues for which the backbone conformation was analysed, and the total number of residues.

Mol	Chain	Analysed	Favoured	Allowed	Outliers	Percentiles	
1	A	371/375 (99%)	363 (98%)	8 (2%)	0	100	100
1	D	369/375 (98%)	362 (98%)	7 (2%)	0	100	100
2	B	258/260 (99%)	251 (97%)	7 (3%)	0	100	100
2	F	258/260 (99%)	252 (98%)	6 (2%)	0	100	100
3	C	36/40 (90%)	36 (100%)	0	0	100	100
3	E	37/40 (92%)	37 (100%)	0	0	100	100
All	All	1329/1350 (98%)	1301 (98%)	28 (2%)	0	100	100

There are no Ramachandran outliers to report.

5.3.2 Protein sidechains (i)

In the following table, the Percentiles column shows the percent sidechain outliers of the chain as a percentile score with respect to all X-ray entries followed by that with respect to entries of similar resolution.

The Analysed column shows the number of residues for which the sidechain conformation was analysed, and the total number of residues.

Mol	Chain	Analysed	Rotameric	Outliers	Percentiles	
1	A	314/318 (99%)	314 (100%)	0	100	100
1	D	313/318 (98%)	311 (99%)	2 (1%)	86	92
2	B	229/229 (100%)	229 (100%)	0	100	100
2	F	229/229 (100%)	228 (100%)	1 (0%)	91	95
3	C	24/32 (75%)	24 (100%)	0	100	100
3	E	26/32 (81%)	26 (100%)	0	100	100
All	All	1135/1158 (98%)	1132 (100%)	3 (0%)	92	96

CONFIDENTIAL VALIDATION REPORT

All (3) residues with a non-rotameric sidechain are listed below:

Mol	Chain	Res	Type
1	D	116	ARG
1	D	297	ASN
2	F	221	LEU

Sometimes sidechains can be flipped to improve hydrogen bonding and reduce clashes. There are no such sidechains identified.

5.3.3 RNA (i)

There are no RNA molecules in this entry.

5.4 Non-standard residues in protein, DNA, RNA chains (i)

There are no non-standard protein/DNA/RNA residues in this entry.

5.5 Carbohydrates (i)

4 monosaccharides are modelled in this entry.

In the following table, the Counts columns list the number of bonds (or angles) for which Mogul statistics could be retrieved, the number of bonds (or angles) that are observed in the model and the number of bonds (or angles) that are defined in the Chemical Component Dictionary. The Link column lists molecule types, if any, to which the group is linked. The Z score for a bond length (or angle) is the number of standard deviations the observed value is removed from the expected value. A bond length (or angle) with $|Z| > 2$ is considered an outlier worth inspection. RMSZ is the root-mean-square of all Z scores of the bond lengths (or angles).

Mol	Type	Chain	Res	Link	Bond lengths			Bond angles		
					Counts	RMSZ	$\#\|Z\| > 2$	Counts	RMSZ	$\#\|Z\| > 2$
4	NAG	G	1	2,4	14,14,15	0.32	0	17,19,21	1.21	1 (5%)
4	NAG	G	2	4	14,14,15	0.31	0	17,19,21	0.95	1 (5%)
4	NAG	H	1	2,4	14,14,15	0.33	0	17,19,21	0.79	0
4	NAG	H	2	4	14,14,15	0.42	0	17,19,21	1.75	4 (23%)

In the following table, the Chirals column lists the number of chiral outliers, the number of chiral centers analysed, the number of these observed in the model and the number defined in the Chemical Component Dictionary. Similar counts are reported in the Torsion and Rings columns. '-' means no outliers of that kind were identified.

CONFIDENTIAL VALIDATION REPORT

Mol	Type	Chain	Res	Link	Chirals	Torsions	Rings
4	NAG	G	1	2,4	-	0/6/23/26	0/1/1/1
4	NAG	G	2	4	-	2/6/23/26	0/1/1/1
4	NAG	H	1	2,4	-	0/6/23/26	0/1/1/1
4	NAG	H	2	4	-	3/6/23/26	0/1/1/1

There are no bond length outliers.

All (6) bond angle outliers are listed below:

Mol	Chain	Res	Type	Atoms	Z	Observed(^{o})	Ideal(^{o})
4	G	1	NAG	C1-O5-C5	4.46	118.24	112.19
4	H	2	NAG	C2-N2-C7	4.20	128.89	122.90
4	H	2	NAG	C8-C7-N2	3.46	121.96	116.10
4	G	2	NAG	C1-O5-C5	3.03	116.30	112.19
4	H	2	NAG	C1-O5-C5	2.95	116.19	112.19
4	H	2	NAG	O5-C5-C6	2.06	110.44	107.20

There are no chirality outliers.

All (5) torsion outliers are listed below:

Mol	Chain	Res	Type	Atoms
4	G	2	NAG	O5-C5-C6-O6
4	H	2	NAG	C8-C7-N2-C2
4	H	2	NAG	O7-C7-N2-C2
4	G	2	NAG	C4-C5-C6-O6
4	H	2	NAG	C3-C2-N2-C7

There are no ring outliers.

1 monomer is involved in 2 short contacts:

Mol	Chain	Res	Type	Clashes	Symm-Clashes
4	H	2	NAG	2	0

The following is a two-dimensional graphical depiction of Mogul quality analysis of bond lengths, bond angles, torsion angles, and ring geometry for oligosaccharide.

CONFIDENTIAL VALIDATION REPORT

Oligosaccharide Chain G

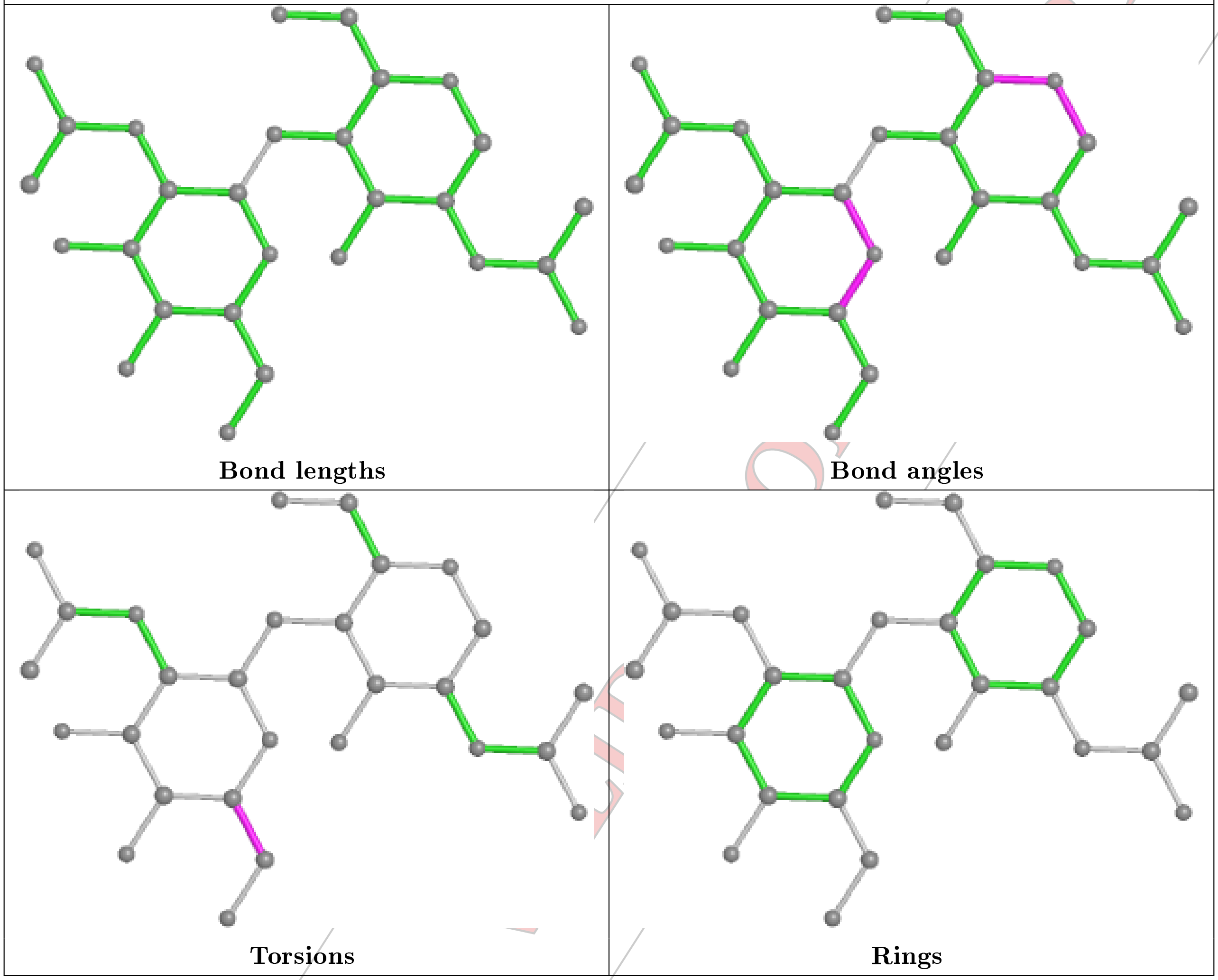

CONFIDENTIAL

Oligosaccharide Chain H

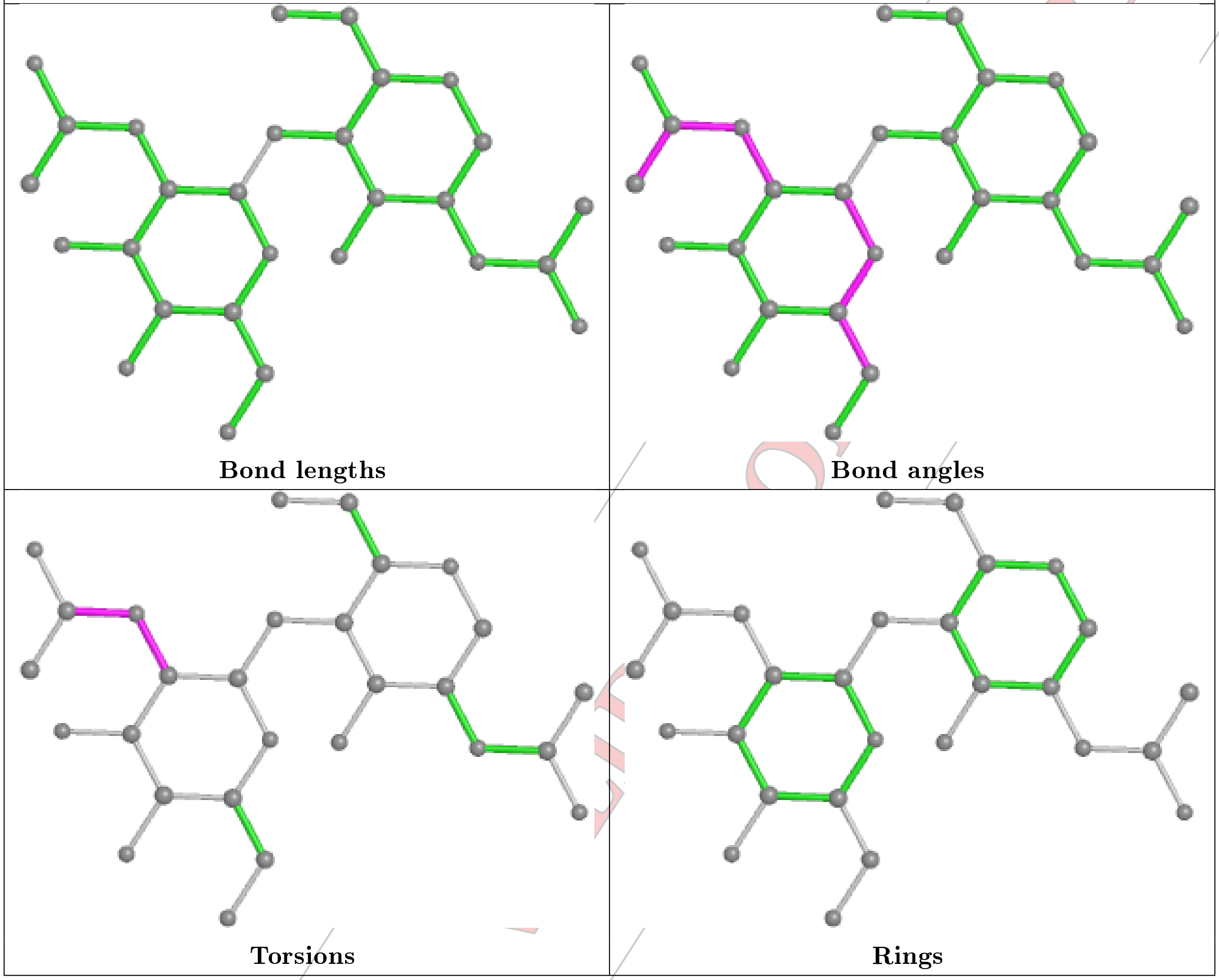

5.6 Ligand geometry ⓘ

Of 8 ligands modelled in this entry, 6 are monoatomic - leaving 2 for Mogul analysis.

In the following table, the Counts columns list the number of bonds (or angles) for which Mogul statistics could be retrieved, the number of bonds (or angles) that are observed in the model and the number of bonds (or angles) that are defined in the Chemical Component Dictionary. The Link column lists molecule types, if any, to which the group is linked. The Z score for a bond length (or angle) is the number of standard deviations the observed value is removed from the expected value. A bond length (or angle) with $|Z| > 2$ is considered an outlier worth inspection. RMSZ is the root-mean-square of all Z scores of the bond lengths (or angles).

Mol	Type	Chain	Res	Link	Bond lengths			Bond angles		
					Counts	RMSZ	$\#\|Z\| > 2$	Counts	RMSZ	$\#\|Z\| > 2$
5	ATP	D	401	6	26,33,33	0.67	0	31,52,52	0.74	1 (3%)
5	ATP	A	401	6	26,33,33	0.66	0	31,52,52	0.76	1 (3%)

In the following table, the Chirals column lists the number of chiral outliers, the number of chiral centers analysed, the number of these observed in the model and the number defined in the Chemical Component Dictionary. Similar counts are reported in the Torsion and Rings columns. '-' means no outliers of that kind were identified.

Mol	Type	Chain	Res	Link	Chirals	Torsions	Rings
5	ATP	D	401	6	-	3/18/38/38	0/3/3/3
5	ATP	A	401	6	-	3/18/38/38	0/3/3/3

There are no bond length outliers.

All (2) bond angle outliers are listed below:

Mol	Chain	Res	Type	Atoms	Z	Observed(^{o})	Ideal(^{o})
5	D	401	ATP	C5-C6-N6	2.20	123.69	120.35
5	A	401	ATP	C5-C6-N6	2.19	123.68	120.35

There are no chirality outliers.

All (6) torsion outliers are listed below:

Mol	Chain	Res	Type	Atoms
5	A	401	ATP	PG-O3B-PB-O1B
5	D	401	ATP	PG-O3B-PB-O1B
5	A	401	ATP	PB-O3B-PG-O1G
5	A	401	ATP	PG-O3B-PB-O2B
5	D	401	ATP	PG-O3B-PB-O2B
5	D	401	ATP	PB-O3B-PG-O1G

There are no ring outliers.

No monomer is involved in short contacts.

The following is a two-dimensional graphical depiction of Mogul quality analysis of bond lengths, bond angles, torsion angles, and ring geometry for all instances of the Ligand of Interest. In addition, ligands with molecular weight > 250 and outliers as shown on the validation Tables will also be included. For torsion angles, if less then 5% of the Mogul distribution of torsion angles is within 10 degrees of the torsion angle in question, then that torsion angle is considered an outlier. Any bond that is central to one or more torsion angles identified as an outlier by Mogul will be highlighted in the graph. For rings, the root-mean-square deviation (RMSD) between the ring in question and similar rings identified by Mogul is calculated over all ring torsion angles. If the average RMSD is greater than 60 degrees and the minimal RMSD between the ring in question and any Mogul-identified rings is also greater than 60 degrees, then that ring is considered an outlier. The outliers are highlighted in purple. The color gray indicates Mogul did not find sufficient equivalents in the CSD to analyse the geometry.

CONFIDENTIAL VALIDATION REPORT

Ligand ATP D 401

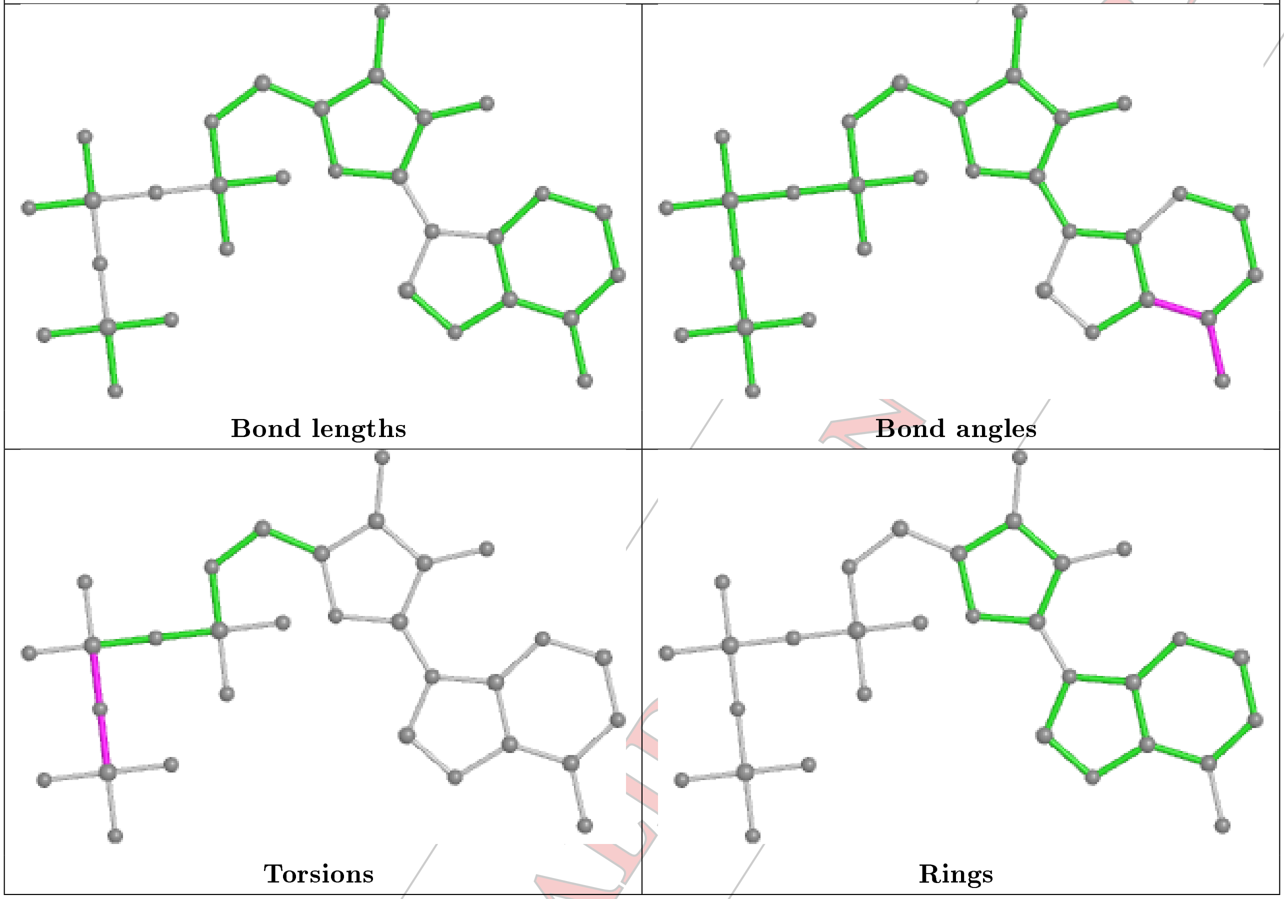

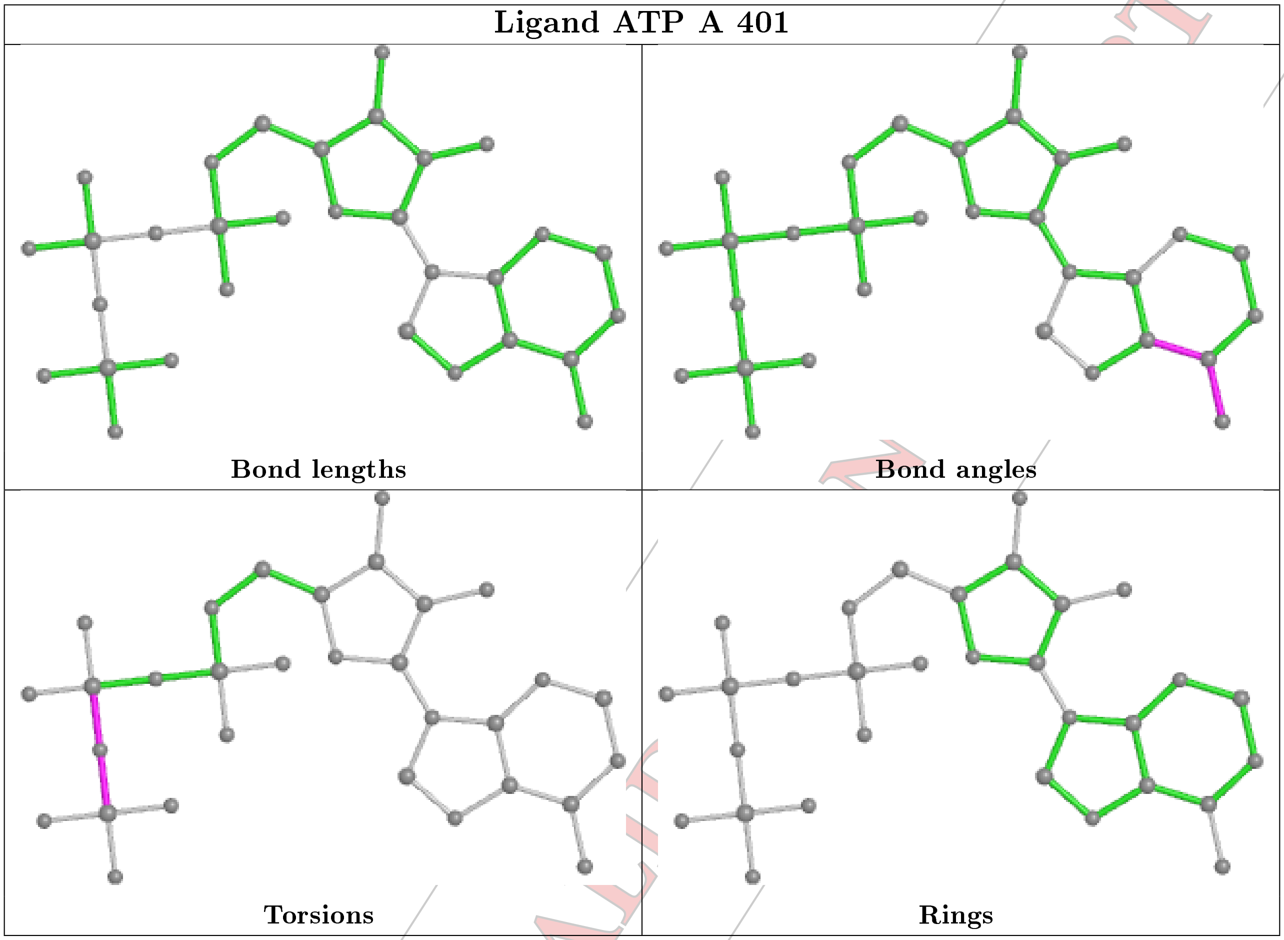

5.7 Other polymers (i)

There are no such residues in this entry.

5.8 Polymer linkage issues (i)

There are no chain breaks in this entry.

CONFIDENTIAL VALIDATION REPORT

6 Fit of model and data (i)

6.1 Protein, DNA and RNA chains (i)

In the following table, the column labelled '#RSRZ> 2' contains the number (and percentage) of RSRZ outliers, followed by percent RSRZ outliers for the chain as percentile scores relative to all X-ray entries and entries of similar resolution. The OWAB column contains the minimum, median, 95th percentile and maximum values of the occupancy-weighted average B-factor per residue. The column labelled 'Q< 0.9' lists the number of (and percentage) of residues with an average occupancy less than 0.9.

Mol	Chain	Analysed	<RSRZ>	#RSRZ>2			OWAB(Å^2)	Q<0.9
1	A	373/375 (99%)	0.20	4 (1%)	80	85	27, 39, 63, 85	0
1	D	371/375 (98%)	0.08	2 (0%)	91	94	27, 38, 52, 62	0
2	B	260/260 (100%)	-0.02	1 (0%)	92	96	27, 33, 43, 54	0
2	F	260/260 (100%)	-0.05	0	100	100	25, 33, 45, 55	0
3	C	38/40 (95%)	0.78	2 (5%)	26	31	41, 56, 74, 77	0
3	E	39/40 (97%)	0.51	2 (5%)	28	33	35, 47, 84, 91	0
All	All	1341/1350 (99%)	0.10	11 (0%)	86	89	25, 36, 58, 91	0

All (11) RSRZ outliers are listed below:

Mol	Chain	Res	Type	RSRZ
1	D	270	GLU	2.8
3	E	585	LEU	2.8
1	A	268	GLY	2.4
1	D	69	TYR	2.3
1	A	199	SER	2.3
3	E	621	ASN	2.2
1	A	323	SER	2.2
2	B	156	GLN	2.1
1	A	166	TYR	2.1
3	C	605	PRO	2.1
3	C	617	ALA	2.0

6.2 Non-standard residues in protein, DNA, RNA chains (i)

There are no non-standard protein/DNA/RNA residues in this entry.

CONFIDENTIAL VALIDATION REPORT

6.3 Carbohydrates (i)

In the following table, the Atoms column lists the number of modelled atoms in the group and the number defined in the chemical component dictionary. The B-factors column lists the minimum, median, 95th percentile and maximum values of B factors of atoms in the group. The column labelled ‘Q< 0.9’ lists the number of atoms with occupancy less than 0.9.

Mol	Type	Chain	Res	Atoms	RSCC	RSR	B-factors($Å^2$)	Q<0.9
4	NAG	H	2	14/15	0.69	0.30	75,79,82,82	0
4	NAG	H	1	14/15	0.82	0.23	52,56,58,63	0
4	NAG	G	2	14/15	0.87	0.20	44,46,48,48	0
4	NAG	G	1	14/15	0.90	0.17	39,40,42,42	0

The following is a graphical depiction of the model fit to experimental electron density for oligosaccharide. Each fit is shown from different orientation to approximate a three-dimensional view.

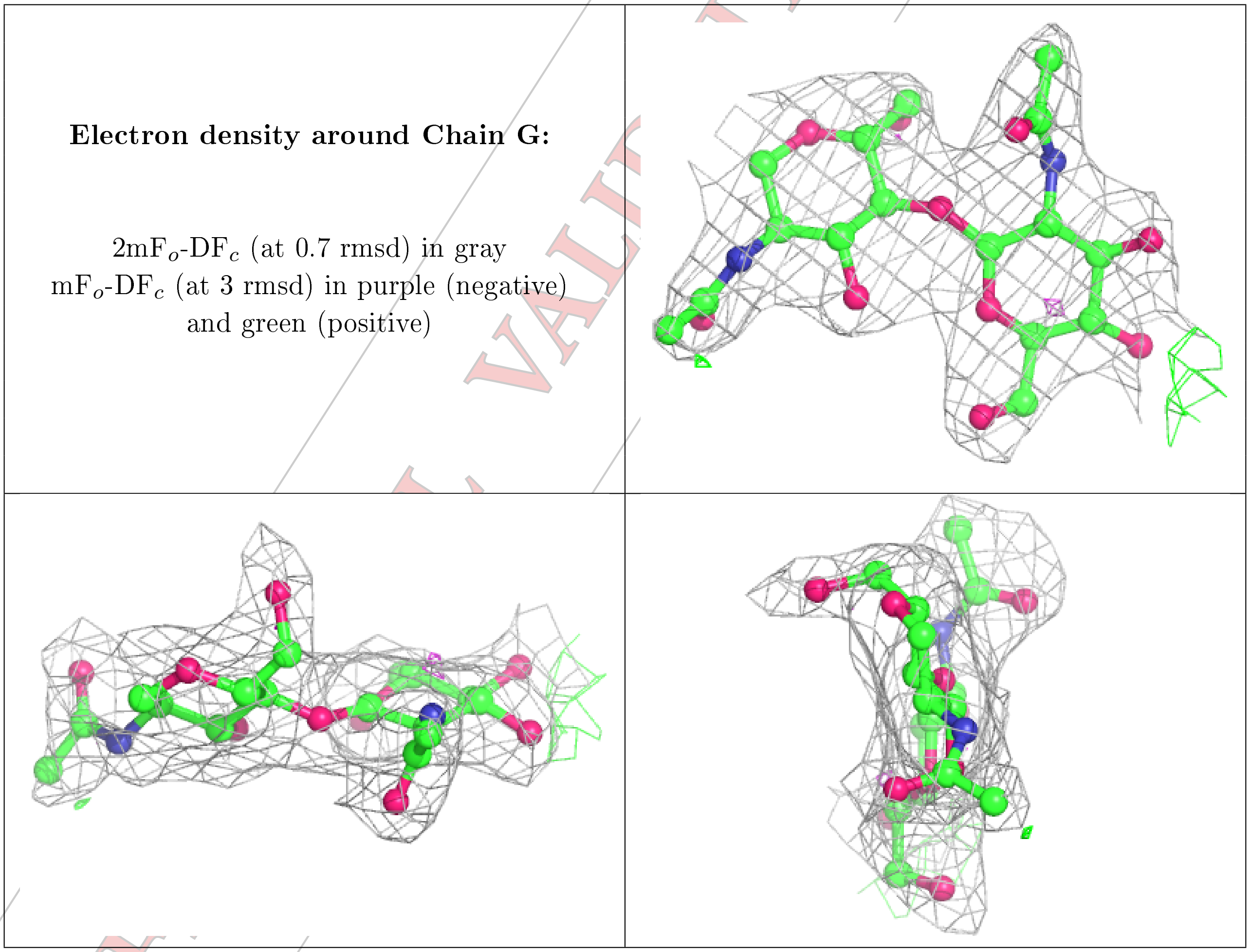

CONFIDENTIAL VALIDATION REPORT

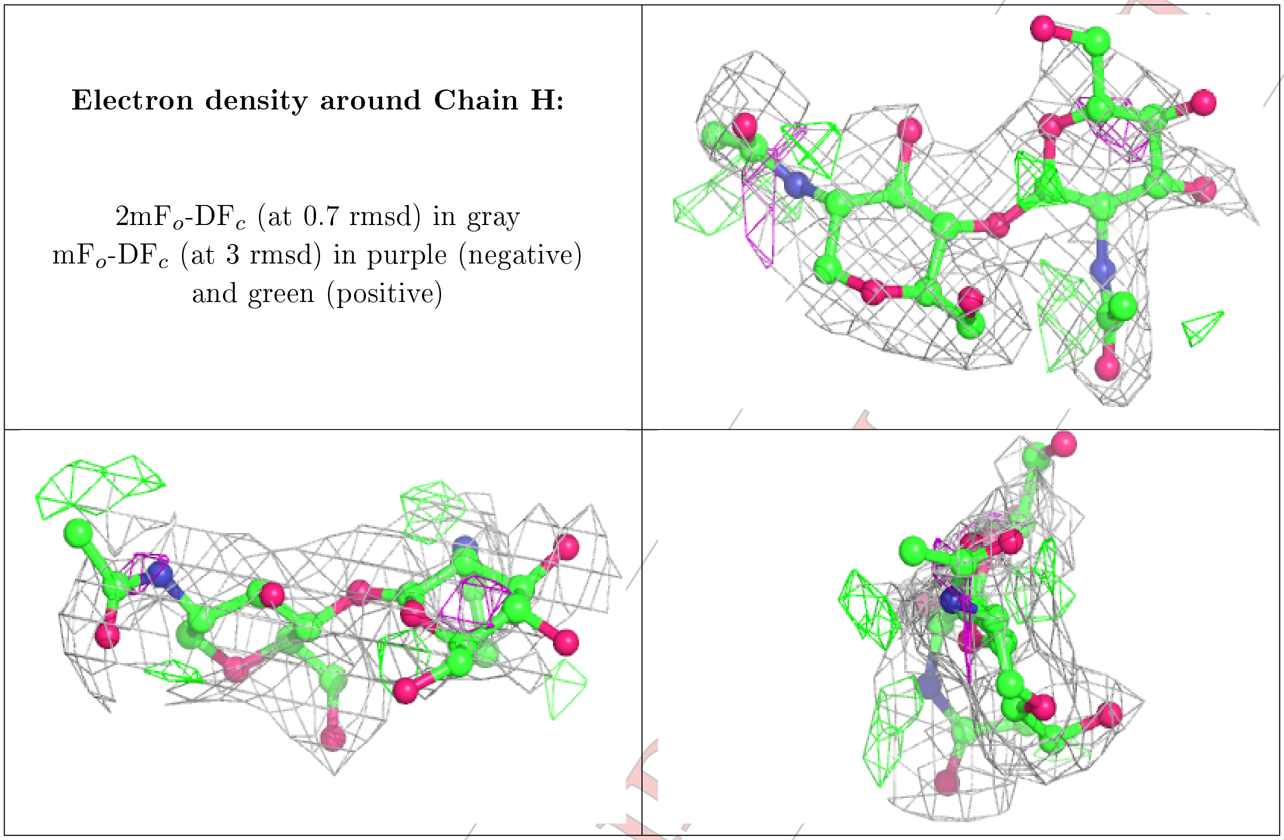

Electron density around Chain H:

2mF$_o$-DF$_c$ (at 0.7 rmsd) in gray
mF$_o$-DF$_c$ (at 3 rmsd) in purple (negative) and green (positive)

6.4 Ligands ⓘ

In the following table, the Atoms column lists the number of modelled atoms in the group and the number defined in the chemical component dictionary. The B-factors column lists the minimum, median, 95th percentile and maximum values of B factors of atoms in the group. The column labelled 'Q< 0.9' lists the number of atoms with occupancy less than 0.9.

Mol	Type	Chain	Res	Atoms	RSCC	RSR	B-factors(Å^2)	Q<0.9
6	CA	F	302	1/1	0.85	0.06	61,61,61,61	0
6	CA	B	302	1/1	0.92	0.09	52,52,52,52	0
6	CA	B	301	1/1	0.93	0.12	42,42,42,42	0
6	CA	F	301	1/1	0.98	0.06	33,33,33,33	0
5	ATP	A	401	31/31	0.98	0.12	25,28,31,32	0
5	ATP	D	401	31/31	0.99	0.12	27,28,29,29	0
6	CA	D	402	1/1	0.99	0.10	27,27,27,27	0
6	CA	A	402	1/1	1.00	0.10	26,26,26,26	0

The following is a graphical depiction of the model fit to experimental electron density of all instances of the Ligand of Interest. In addition, ligands with molecular weight > 250 and outliers as shown on the geometry validation Tables will also be included. Each fit is shown from different orientation to approximate a three-dimensional view.

Electron density around ATP A 401:

$2mF_o$-DF_c (at 0.7 rmsd) in gray
mF_o-DF_c (at 3 rmsd) in purple (negative)
and green (positive)

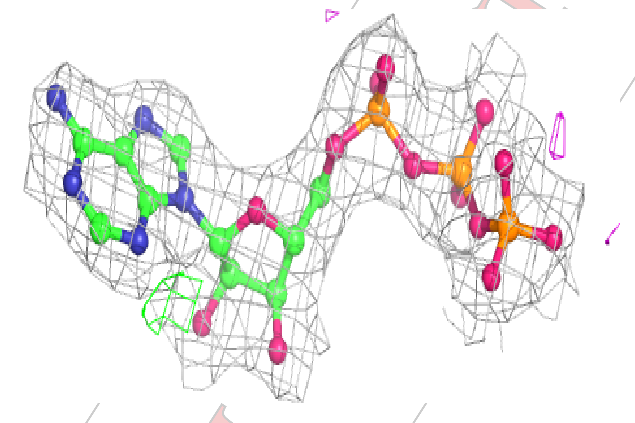

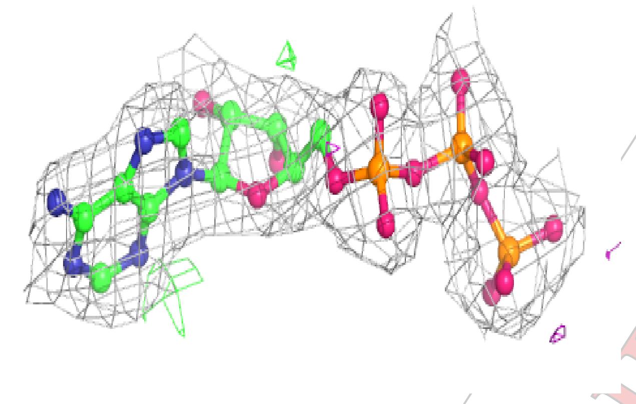

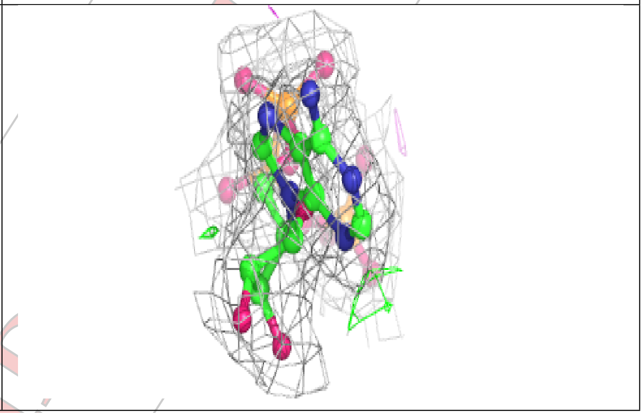

Electron density around ATP D 401:

$2mF_o$-DF_c (at 0.7 rmsd) in gray
mF_o-DF_c (at 3 rmsd) in purple (negative)
and green (positive)

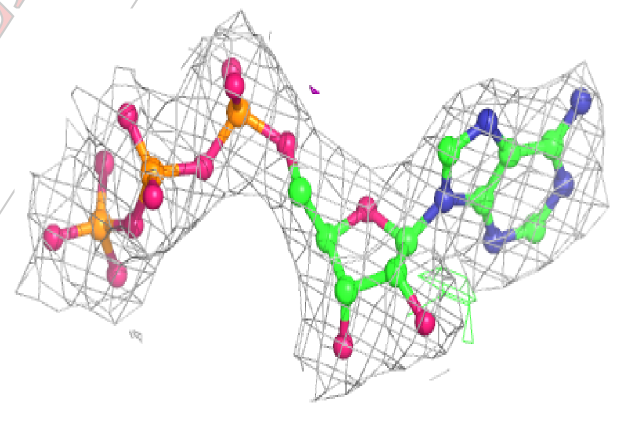

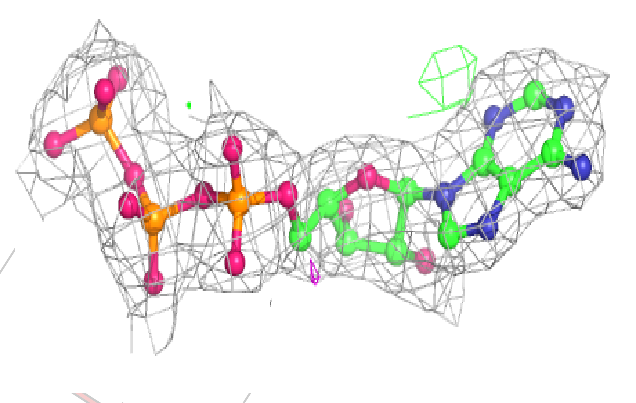

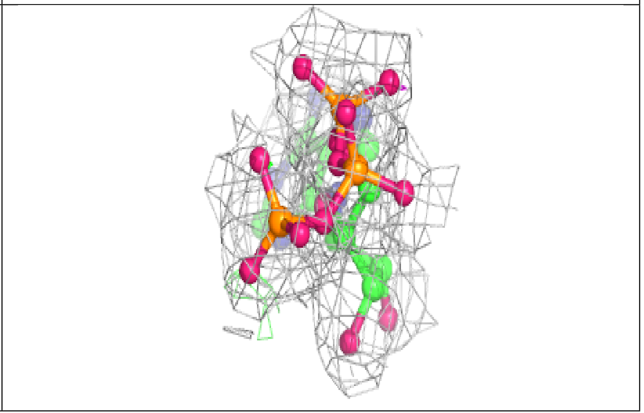

6.5 Other polymers ⓘ

There are no such residues in this entry.

CONFIDENTIAL VALIDATION REPORT

Full wwPDB EM Validation Report (i)

Mar 26, 2021 – 03:33 pm GMT

PDB ID	:	7NZM
EMDB ID	:	EMD-12665
Title	:	Cryo-EM structure of pre-dephosphorylation complex of phosphorylated eIF2alpha with trapped holophosphatase (PP1A_D64A/PPP1R15A/G-actin /DNase I)
Deposited on	:	2021-03-24
Resolution	:	3.96 Å(reported)
Based on initial models	:	2A42, 4MOV, 1KL9

This is a Full wwPDB EM Validation Report.

This report is produced by the wwPDB biocuration pipeline after annotation of the structure.

We welcome your comments at *validation@mail.wwpdb.org*
A user guide is available at
https://www.wwpdb.org/validation/2017/EMValidationReportHelp
with specific help available everywhere you see the (i) symbol.

The following versions of software and data (see references (i)) were used in the production of this report:

EMDB validation analysis	:	0.0.0.dev75
Mogul	:	1.8.5 (274361), CSD as541be (2020)
MolProbity	:	4.02b-467
buster-report	:	1.1.7 (2018)
Percentile statistics	:	20191225.v01 (using entries in the PDB archive December 25th 2019)
Ideal geometry (proteins)	:	Engh & Huber (2001)
Ideal geometry (DNA, RNA)	:	Parkinson et al. (1996)
Validation Pipeline (wwPDB-VP)	:	2.18

CONFIDENTIAL VALIDATION REPORT

1 Overall quality at a glance (i)

The following experimental techniques were used to determine the structure:
ELECTRON MICROSCOPY

The reported resolution of this entry is 3.96 Å.

Percentile scores (ranging between 0-100) for global validation metrics of the entry are shown in the following graphic. The table shows the number of entries on which the scores are based.

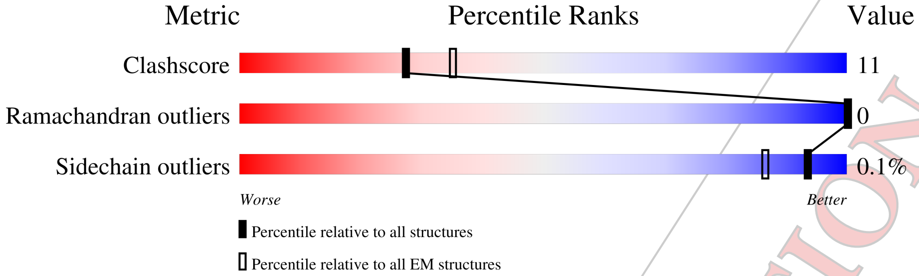

Metric	Whole archive (#Entries)	EM structures (#Entries)
Clashscore	158937	4297
Ramachandran outliers	154571	4023
Sidechain outliers	154315	3826

The table below summarises the geometric issues observed across the polymeric chains and their fit to the map. The red, orange, yellow and green segments of the bar indicate the fraction of residues that contain outliers for >=3, 2, 1 and 0 types of geometric quality criteria respectively. A grey segment represents the fraction of residues that are not modelled. The numeric value for each fraction is indicated below the corresponding segment, with a dot representing fractions <=5% The upper red bar (where present) indicates the fraction of residues that have poor fit to the EM map (all-atom inclusion < 40%). The numeric value is given above the bar.

Mol	Chain	Length	Quality of chain
1	E	186	12%<br>73% 22% • •
2	B	294	16%<br>68% 32%
3	A	375	•<br>82% 16% •
4	D	260	11%<br>74% 24% •
5	C	444	•<br>10% • 87%
6	F	2	50%<br>50% 50%

CONFIDENTIAL VALIDATION REPORT

2 Entry composition (i)

There are 8 unique types of molecules in this entry. The entry contains 9109 atoms, of which 0 are hydrogens and 0 are deuteriums.

In the tables below, the AltConf column contains the number of residues with at least one atom in alternate conformation and the Trace column contains the number of residues modelled with at most 2 atoms.

- Molecule 1 is a protein called Eukaryotic translation initiation factor 2 subunit 1.

Mol	Chain	Residues	Atoms						AltConf	Trace
			Total	C	N	O	P	S		
1	E	178	1392	887	232	267	1	5	0	0

- Molecule 2 is a protein called Serine/threonine-protein phosphatase PP1-alpha catalytic subunit.

Mol	Chain	Residues	Atoms					AltConf	Trace
			Total	C	N	O	S		
2	B	293	2312	1483	387	424	18	0	0

There is a discrepancy between the modelled and reference sequences:

Chain	Residue	Modelled	Actual	Comment	Reference
B	64	ALA	ASP	engineered mutation	UNP P62139

- Molecule 3 is a protein called Actin, alpha skeletal muscle, intermediate form.

Mol	Chain	Residues	Atoms					AltConf	Trace
			Total	C	N	O	S		
3	A	370	2884	1827	487	549	21	0	0

- Molecule 4 is a protein called Deoxyribonuclease-1.

Mol	Chain	Residues	Atoms					AltConf	Trace
			Total	C	N	O	S		
4	D	257	2024	1282	337	397	8	0	0

- Molecule 5 is a protein called Protein phosphatase 1 regulatory subunit 15A,Maltose/maltodextrin-binding periplasmic protein.

Mol	Chain	Residues	Atoms					AltConf	Trace
			Total	C	N	O	S		
5	C	59	437	271	88	77	1	0	0

CONFIDENTIAL VALIDATION REPORT

There is a discrepancy between the modelled and reference sequences:

Chain	Residue	Modelled	Actual	Comment	Reference
C	625	GLN	-	linker	UNP O75807

- Molecule 6 is an oligosaccharide called 2-acetamido-2-deoxy-beta-D-glucopyranose-(1-4)-2-acetamido-2-deoxy-beta-D-glucopyranose.

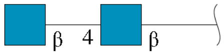

Mol	Chain	Residues	Atoms	AltConf	Trace
6	F	2	Total C N O<br>28 16 2 10	0	0

- Molecule 7 is MANGANESE (II) ION (three-letter code: MN) (formula: Mn) (labeled as "Ligand of Interest" by depositor).

Mol	Chain	Residues	Atoms	AltConf
7	B	1	Total Mn<br>1 1	0

- Molecule 8 is ADENOSINE-5'-TRIPHOSPHATE (three-letter code: ATP) (formula: $C_{10}H_{16}N_5O_{13}P_3$).

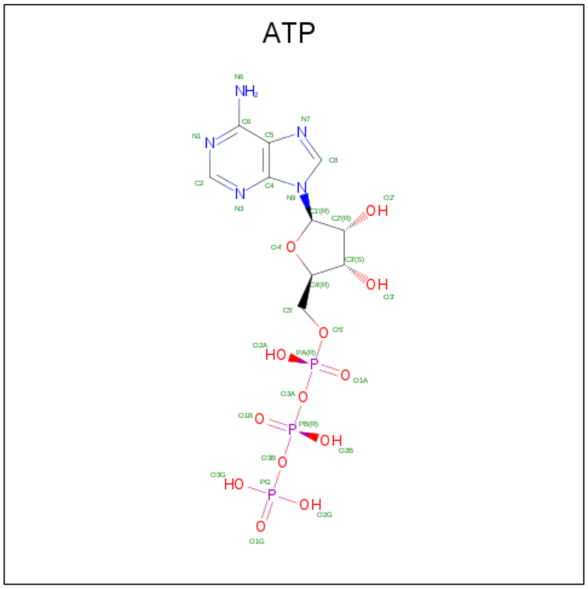

CONFIDENTIAL VALIDATION REPORT

Mol	Chain	Residues	Atoms					AltConf
			Total	C	N	O	P	
8	A	1	31	10	5	13	3	0

CONFIDENTIAL VALIDATION REPORT

3 Residue-property plots (i)

These plots are drawn for all protein, RNA, DNA and oligosaccharide chains in the entry. The first graphic for a chain summarises the proportions of the various outlier classes displayed in the second graphic. The second graphic shows the sequence view annotated by issues in geometry and atom inclusion in map density. Residues are color-coded according to the number of geometric quality criteria for which they contain at least one outlier: green = 0, yellow = 1, orange = 2 and red = 3 or more. A red diamond above a residue indicates a poor fit to the EM map for this residue (all-atom inclusion < 40%). Stretches of 2 or more consecutive residues without any outlier are shown as a green connector. Residues present in the sample, but not in the model, are shown in grey.

- Molecule 1: Eukaryotic translation initiation factor 2 subunit 1

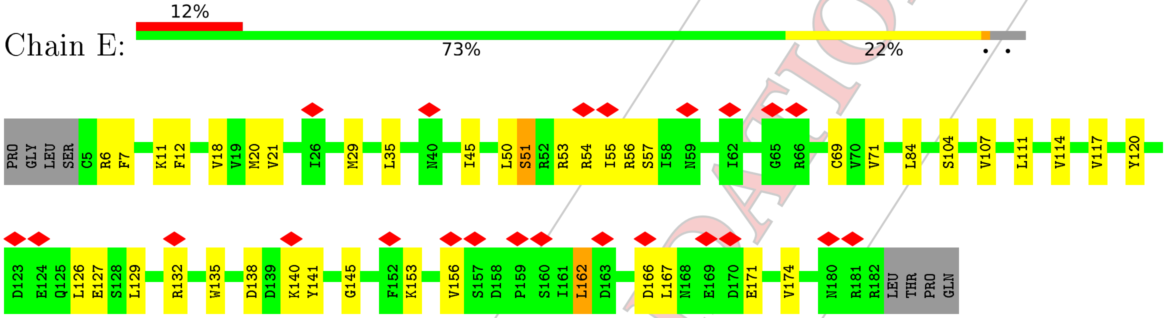

- Molecule 2: Serine/threonine-protein phosphatase PP1-alpha catalytic subunit

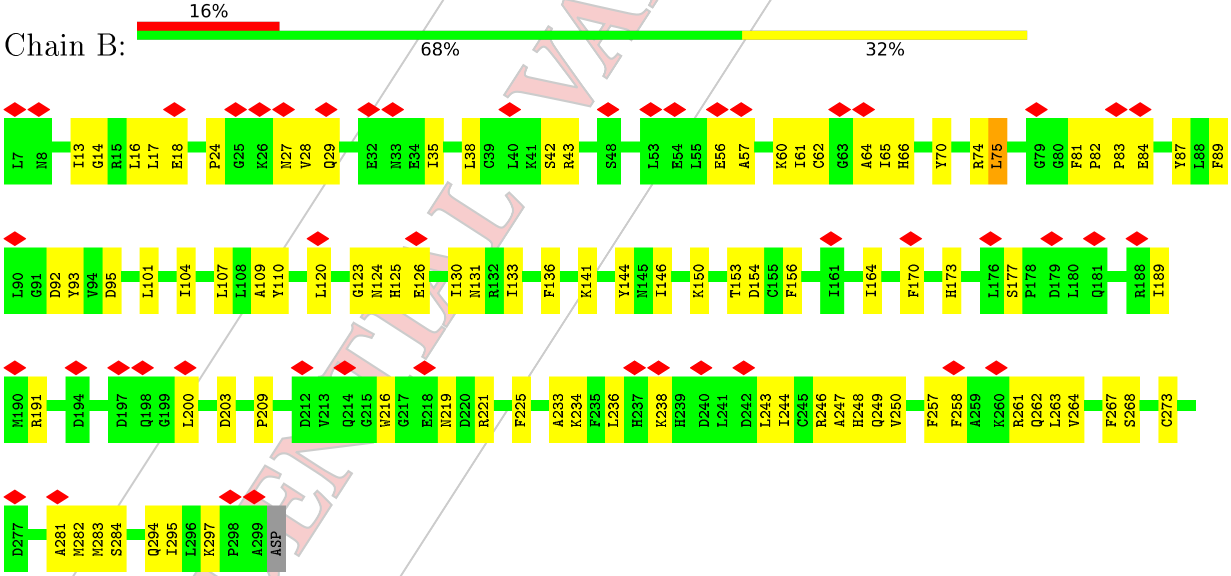

- Molecule 3: Actin, alpha skeletal muscle, intermediate form

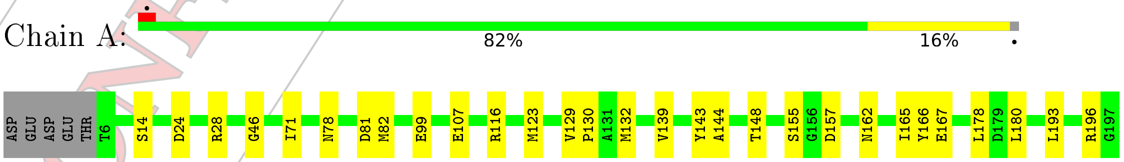

CONFIDENTIAL VALIDATION REPORT

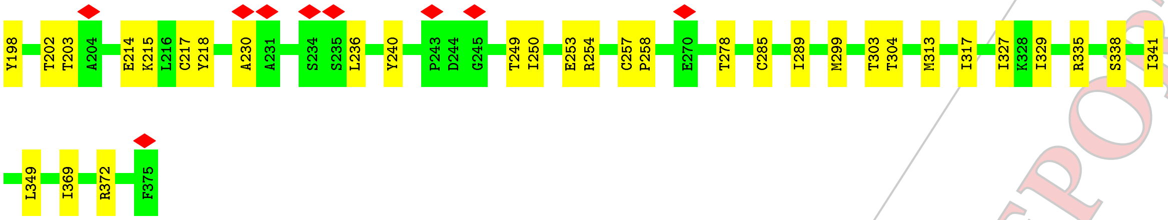

• Molecule 4: Deoxyribonuclease-1

Chain D: 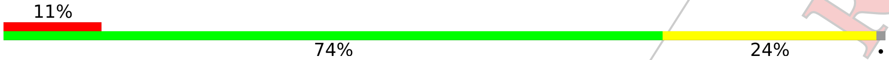

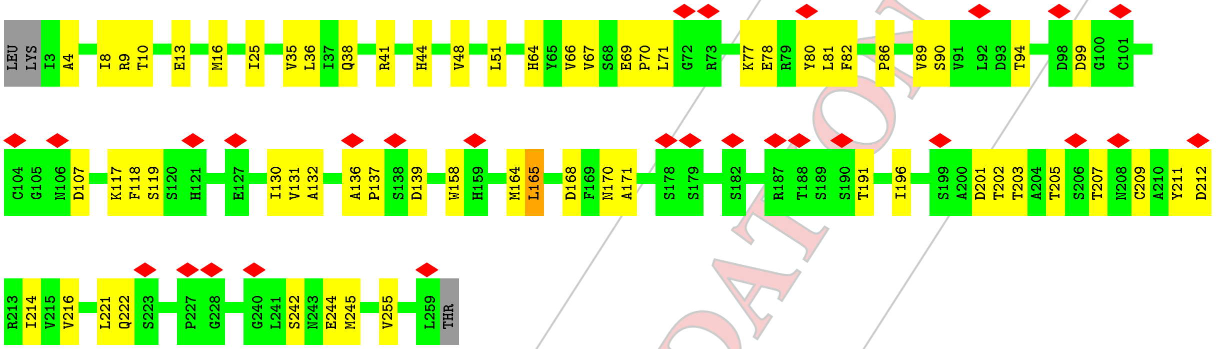

• Molecule 5: Protein phosphatase 1 regulatory subunit 15A,Maltose/maltodextrin-binding periplasmic protein

Chain C: 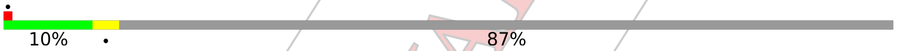

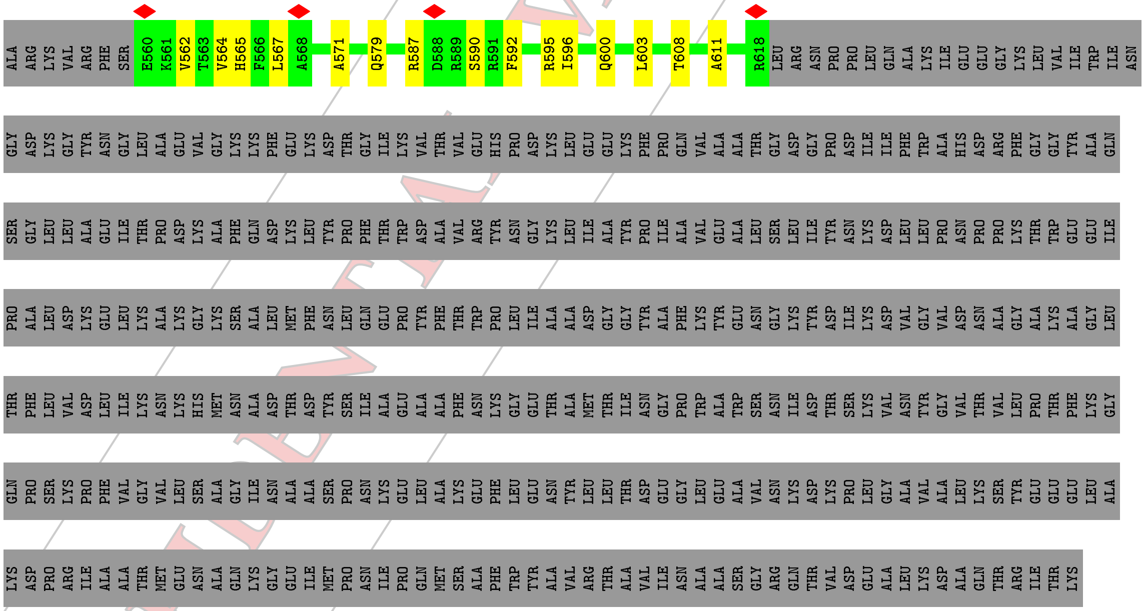

• Molecule 6: 2-acetamido-2-deoxy-beta-D-glucopyranose-(1-4)-2-acetamido-2-deoxy-beta-D-gluc opyranose

Chain F: 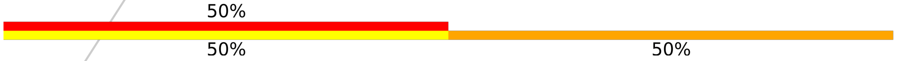

CONFIDENTIAL VALIDATION REPORT

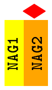

CONFIDENTIAL VALIDATION REPORT

4 Experimental information (i)

Property	Value	Source
EM reconstruction method	SINGLE PARTICLE	Depositor
Imposed symmetry	POINT, C1	Depositor
Number of particles used	60413	Depositor
Resolution determination method	FSC 0.143 CUT-OFF	Depositor
CTF correction method	PHASE FLIPPING AND AMPLITUDE CORRECTION; Warp estimated the CTF parameters and passed them on to CryoSPARC to perform CTF correction.	Depositor
Microscope	FEI TITAN KRIOS	Depositor
Voltage (kV)	300	Depositor
Electron dose $(e^-/\text{Å}^2)$	46.84	Depositor
Minimum defocus (nm)	-1000	Depositor
Maximum defocus (nm)	-2800	Depositor
Magnification	130000	Depositor
Image detector	GATAN K3 BIOQUANTUM (6k x 4k)	Depositor
Maximum map value	0.166	Depositor
Minimum map value	-0.031	Depositor
Average map value	0.001	Depositor
Map value standard deviation	0.007	Depositor
Recommended contour level	0.05	Depositor
Map size (Å)	221.68001, 221.68001, 221.68001	wwPDB
Map dimensions	340, 340, 340	wwPDB
Map angles (°)	90.0, 90.0, 90.0	wwPDB
Pixel spacing (Å)	0.652, 0.652, 0.652	Depositor

CONFIDENTIAL VALIDATION REPORT

5 Model quality (i)

5.1 Standard geometry (i)

Bond lengths and bond angles in the following residue types are not validated in this section: SEP, ATP, NAG, MN

The Z score for a bond length (or angle) is the number of standard deviations the observed value is removed from the expected value. A bond length (or angle) with $|Z| > 5$ is considered an outlier worth inspection. RMSZ is the root-mean-square of all Z scores of the bond lengths (or angles).

Mol	Chain	Bond lengths		Bond angles	
		RMSZ	#|Z| >5	RMSZ	#|Z| >5
1	E	0.36	0/1404	0.61	1/1899 (0.1%)
2	B	0.34	0/2366	0.67	1/3204 (0.0%)
3	A	0.38	0/2947	0.56	0/3993
4	D	0.35	0/2070	0.59	1/2821 (0.0%)
5	C	0.31	0/446	0.47	0/608
All	All	0.36	0/9233	0.60	3/12525 (0.0%)

There are no bond length outliers.

All (3) bond angle outliers are listed below:

Mol	Chain	Res	Type	Atoms	Z	Observed(°)	Ideal(°)
4	D	165	LEU	CA-CB-CG	6.04	129.20	115.30
1	E	162	LEU	CA-CB-CG	5.73	128.48	115.30
2	B	75	LEU	CA-CB-CG	5.65	128.29	115.30

There are no chirality outliers.

There are no planarity outliers.

5.2 Too-close contacts (i)

In the following table, the Non-H and H(model) columns list the number of non-hydrogen atoms and hydrogen atoms in the chain respectively. The H(added) column lists the number of hydrogen atoms added and optimized by MolProbity. The Clashes column lists the number of clashes within the asymmetric unit, whereas Symm-Clashes lists symmetry-related clashes.

Mol	Chain	Non-H	H(model)	H(added)	Clashes	Symm-Clashes
1	E	1392	0	1325	34	0
2	B	2312	0	2234	72	0
3	A	2884	0	2849	46	0

Continued on next page...

CONFIDENTIAL VALIDATION REPORT

Continued from previous page...

Mol	Chain	Non-H	H(model)	H(added)	Clashes	Symm-Clashes
4	D	2024	0	1949	45	0
5	C	437	0	420	18	0
6	F	28	0	25	1	0
7	B	1	0	0	0	0
8	A	31	0	12	4	0
All	All	9109	0	8814	194	0

The all-atom clashscore is defined as the number of clashes found per 1000 atoms (including hydrogen atoms). The all-atom clashscore for this structure is 11.

All (194) close contacts within the same asymmetric unit are listed below, sorted by their clash magnitude.

Atom-1	Atom-2	Interatomic distance (Å)	Clash overlap (Å)
3:A:250:ILE:HD11	3:A:253:GLU:HB2	1.50	0.92
3:A:299:MET:HB3	3:A:304:THR:HG21	1.60	0.84
2:B:95:ASP:OD2	2:B:125:HIS:ND1	2.13	0.81
2:B:60:LYS:NZ	2:B:284:SER:OG	2.18	0.75
2:B:131:ASN:HB2	2:B:136:PHE:HB3	1.69	0.73
4:D:202:THR:HG21	4:D:212:ASP:H	1.53	0.72
1:E:6:ARG:NH2	1:E:127:GLU:OE2	2.23	0.72
4:D:196:ILE:HD13	4:D:214:ILE:H	1.53	0.71
2:B:243:LEU:HD13	2:B:262:GLN:HA	1.72	0.70
4:D:38:GLN:HE22	4:D:168:ASP:H	1.40	0.68
1:E:53:ARG:O	1:E:57:SER:OG	2.12	0.68
4:D:242:SER:OG	4:D:244:GLU:OE1	2.12	0.68
2:B:282:MET:HB2	2:B:294:GLN:HB2	1.76	0.67
4:D:131:VAL:HB	4:D:165:LEU:HD13	1.76	0.67
2:B:209:PRO:HG3	2:B:246:ARG:HH12	1.62	0.65
5:C:565:HIS:HB3	5:C:567:LEU:HD13	1.79	0.65
2:B:38:LEU:O	2:B:42:SER:OG	2.11	0.65
1:E:114:VAL:HG12	1:E:129:LEU:HD21	1.77	0.64
3:A:144:ALA:O	5:C:595:ARG:NH1	2.32	0.63
2:B:282:MET:O	2:B:294:GLN:N	2.30	0.62
2:B:257:PHE:CD1	2:B:261:ARG:HA	2.34	0.62
2:B:75:LEU:HG	2:B:282:MET:SD	2.40	0.60
1:E:117:VAL:HG11	1:E:174:VAL:HG11	1.83	0.59
1:E:132:ARG:HD2	1:E:166:ASP:HB3	1.84	0.59
3:A:338:SER:HA	3:A:341:ILE:HG22	1.83	0.59
4:D:205:THR:HG23	4:D:207:THR:H	1.67	0.59
3:A:236:LEU:O	3:A:254:ARG:NH1	2.35	0.59
2:B:126:GLU:OE1	2:B:126:GLU:N	2.36	0.58

Continued on next page...

CONFIDENTIAL VALIDATION REPORT

Continued from previous page...

Atom-1	Atom-2	Interatomic distance (Å)	Clash overlap (Å)
3:A:107:GLU:OE1	3:A:116:ARG:NE	2.36	0.58
4:D:245:MET:SD	6:F:2:NAG:H81	2.43	0.58
3:A:24:ASP:OD2	3:A:28:ARG:NH1	2.36	0.58
3:A:143:TYR:HB3	5:C:603:LEU:HD21	1.85	0.58
2:B:35:ILE:HA	2:B:38:LEU:HD13	1.86	0.58
4:D:69:GLU:O	4:D:71:LEU:HD12	2.03	0.57
4:D:164:MET:HB2	4:D:221:LEU:HD11	1.86	0.57
1:E:138:ASP:OD1	1:E:145:GLY:N	2.36	0.57
2:B:62:CYS:SG	2:B:75:LEU:HD11	2.43	0.57
2:B:65:ILE:HD11	2:B:89:PHE:CD2	2.39	0.57
2:B:24:PRO:HA	2:B:70:TYR:CZ	2.39	0.57
1:E:53:ARG:HG3	1:E:54:ARG:H	1.69	0.57
3:A:78:ASN:ND2	3:A:81:ASP:OD1	2.37	0.57
4:D:242:SER:HB3	4:D:245:MET:CE	2.34	0.57
2:B:189:ILE:HD11	2:B:200:LEU:HD23	1.87	0.57
2:B:243:LEU:HB3	2:B:262:GLN:O	2.04	0.57
3:A:157:ASP:OD1	8:A:401:ATP:O3'	2.18	0.56
2:B:264:VAL:HG11	2:B:283:MET:HE2	1.87	0.56
1:E:51:SEP:O3P	2:B:124:ASN:ND2	2.38	0.56
1:E:18:VAL:HG12	1:E:71:VAL:HB	1.88	0.55
2:B:295:ILE:O	5:C:565:HIS:HB2	2.06	0.55
1:E:50:LEU:HD23	2:B:221:ARG:HH22	1.72	0.55
3:A:257:CYS:SG	3:A:258:PRO:HD3	2.47	0.55
2:B:234:LYS:O	2:B:238:LYS:HG3	2.07	0.54
2:B:295:ILE:HD12	5:C:562:VAL:HG13	1.89	0.54
3:A:193:LEU:HD21	3:A:250:ILE:HD13	1.89	0.54
4:D:38:GLN:NE2	4:D:168:ASP:H	2.05	0.54
2:B:219:ASN:HB2	2:B:225:PHE:C	2.28	0.53
3:A:46:GLY:H	4:D:94:THR:HG23	1.73	0.53
4:D:9:ARG:HG3	4:D:9:ARG:HH11	1.73	0.53
4:D:48:VAL:HG22	4:D:81:LEU:HD12	1.89	0.53
4:D:117:LYS:HB2	4:D:158:TRP:CH2	2.42	0.53
1:E:51:SEP:O2P	2:B:125:HIS:NE2	2.42	0.53
2:B:297:LYS:HE3	5:C:564:VAL:HB	1.90	0.52
1:E:29:MET:HG2	2:B:133:ILE:HG23	1.90	0.52
2:B:219:ASN:HB2	2:B:225:PHE:CA	2.40	0.52
4:D:118:PHE:CE2	4:D:130:ILE:HD12	2.44	0.52
3:A:99:GLU:HA	3:A:130:PRO:HD3	1.92	0.52
3:A:304:THR:O	3:A:304:THR:HG22	2.10	0.52
3:A:14:SER:HA	3:A:71:ILE:HB	1.91	0.52
4:D:66:VAL:HB	4:D:82:PHE:HB2	1.92	0.52

Continued on next page...

CONFIDENTIAL VALIDATION REPORT

Continued from previous page...

Atom-1	Atom-2	Interatomic distance (Å)	Clash overlap (Å)
2:B:81:PHE:O	2:B:84:GLU:HG2	2.10	0.51
4:D:136:ALA:HB3	4:D:139:ASP:HB2	1.92	0.51
1:E:7:PHE:CD2	1:E:104:SER:HB3	2.45	0.51
4:D:70:PRO:HB2	4:D:77:LYS:HD3	1.92	0.51
3:A:196:ARG:NH1	3:A:249:THR:O	2.43	0.51
2:B:246:ARG:HH11	2:B:263:LEU:HD21	1.75	0.51
1:E:162:LEU:HD23	1:E:162:LEU:O	2.11	0.51
1:E:153:LYS:O	1:E:156:VAL:HG22	2.11	0.50
1:E:20:MET:HA	1:E:69:CYS:HA	1.94	0.50
4:D:201:ASP:OD1	4:D:203:THR:HG22	2.12	0.50
3:A:196:ARG:HD3	3:A:198:TYR:HE2	1.76	0.50
2:B:70:TYR:O	2:B:74:ARG:HG3	2.11	0.49
3:A:139:VAL:HA	3:A:165:ILE:HD13	1.93	0.49
4:D:8:ILE:HD13	4:D:25:ILE:HD11	1.94	0.49
3:A:215:LYS:HZ1	3:A:240:TYR:HE2	1.60	0.49
2:B:246:ARG:HD2	2:B:247:ALA:N	2.27	0.49
3:A:349:LEU:HD22	5:C:600:GLN:HG3	1.93	0.49
1:E:120:TYR:CD1	1:E:126:LEU:HA	2.47	0.49
3:A:230:ALA:HA	3:A:236:LEU:HD23	1.94	0.49
2:B:107:LEU:HB3	2:B:120:LEU:HD11	1.93	0.49
4:D:242:SER:HB3	4:D:245:MET:HE2	1.95	0.49
4:D:130:ILE:HG12	4:D:164:MET:HE1	1.95	0.48
4:D:4:ALA:HB3	4:D:35:VAL:HG23	1.94	0.48
1:E:114:VAL:CG1	1:E:129:LEU:HD21	2.44	0.48
2:B:281:ALA:HA	2:B:294:GLN:O	2.14	0.48
4:D:196:ILE:HD13	4:D:214:ILE:N	2.24	0.48
2:B:258:PHE:CE1	2:B:263:LEU:HB3	2.49	0.47
1:E:167:LEU:HD23	1:E:171:GLU:HB3	1.96	0.47
2:B:16:LEU:HD23	2:B:28:VAL:HG11	1.95	0.47
4:D:10:THR:HB	4:D:41:ARG:NH2	2.30	0.47
2:B:61:ILE:CG2	2:B:283:MET:HB2	2.44	0.47
3:A:349:LEU:HD23	5:C:596:ILE:HG23	1.96	0.47
4:D:4:ALA:HA	4:D:255:VAL:O	2.15	0.47
4:D:171:ALA:O	4:D:211:TYR:HA	2.14	0.47
2:B:27:ASN:HB3	2:B:29:GLN:OE1	2.15	0.47
4:D:191:THR:O	4:D:222:GLN:NE2	2.47	0.47
2:B:56:GLU:HG3	2:B:57:ALA:O	2.14	0.47
2:B:209:PRO:HD3	2:B:246:ARG:HH22	1.80	0.47
4:D:90:SER:HB2	4:D:119:SER:HB3	1.97	0.47
2:B:150:LYS:O	2:B:153:THR:HG22	2.15	0.47
3:A:202:THR:HA	4:D:13:GLU:OE1	2.15	0.46

Continued on next page...

CONFIDENTIAL VALIDATION REPORT

Continued from previous page...

Atom-1	Atom-2	Interatomic distance (Å)	Clash overlap (Å)
4:D:99:ASP:OD1	4:D:107:ASP:HB2	2.14	0.46
2:B:257:PHE:HD1	2:B:261:ARG:HA	1.81	0.46
2:B:273:CYS:SG	5:C:579:GLN:HA	2.56	0.46
4:D:64:HIS:CD2	4:D:86:PRO:HG3	2.52	0.45
4:D:137:PRO:HA	4:D:170:ASN:ND2	2.31	0.45
4:D:16:MET:CE	4:D:51:LEU:HD13	2.46	0.45
5:C:608:THR:HG23	5:C:611:ALA:H	1.81	0.45
3:A:214:GLU:HA	8:A:401:ATP:N1	2.31	0.45
3:A:214:GLU:HG2	8:A:401:ATP:C4	2.52	0.45
3:A:217:CYS:HG	3:A:258:PRO:HD3	1.82	0.45
1:E:107:VAL:O	1:E:111:LEU:HD23	2.17	0.45
3:A:217:CYS:SG	3:A:258:PRO:HD3	2.56	0.45
4:D:36:LEU:HD11	4:D:132:ALA:HB2	1.98	0.45
1:E:29:MET:HE1	5:C:587:ARG:HA	1.98	0.45
2:B:24:PRO:HD3	5:C:571:ALA:HB1	1.99	0.44
2:B:101:LEU:HD13	2:B:144:TYR:CE2	2.52	0.44
2:B:258:PHE:HE1	2:B:263:LEU:HD13	1.81	0.44
3:A:71:ILE:HD11	3:A:82:MET:SD	2.56	0.44
4:D:78:GLU:N	4:D:78:GLU:OE1	2.50	0.44
2:B:82:PRO:HA	2:B:83:PRO:HA	1.61	0.44
2:B:141:LYS:HB2	2:B:146:ILE:HD11	2.00	0.44
2:B:177:SER:HA	2:B:225:PHE:HZ	1.81	0.44
1:E:7:PHE:O	1:E:135:TRP:NE1	2.31	0.44
2:B:14:GLY:O	2:B:18:GLU:HG3	2.18	0.44
4:D:164:MET:HG2	4:D:216:VAL:HG12	2.00	0.44
1:E:120:TYR:HD1	1:E:126:LEU:HA	1.83	0.44
1:E:7:PHE:HD2	1:E:104:SER:HB3	1.82	0.43
3:A:214:GLU:HG2	8:A:401:ATP:C5	2.53	0.43
2:B:203:ASP:CG	2:B:225:PHE:HE1	2.21	0.43
3:A:317:ILE:HG22	3:A:327:ILE:HD13	2.00	0.43
3:A:166:TYR:CD1	3:A:167:GLU:HG2	2.54	0.43
3:A:217:CYS:SG	3:A:218:TYR:N	2.91	0.43
3:A:123:MET:HE3	3:A:129:VAL:HG11	2.01	0.43
5:C:603:LEU:HD12	5:C:603:LEU:HA	1.77	0.43
2:B:17:LEU:HD11	2:B:110:TYR:OH	2.18	0.43
2:B:64:ALA:O	2:B:268:SER:HB2	2.19	0.43
2:B:123:GLY:HA2	2:B:173:HIS:CD2	2.52	0.43
1:E:11:LYS:C	1:E:12:PHE:HD1	2.21	0.43
1:E:55:ILE:HG23	1:E:56:ARG:H	1.83	0.43
3:A:285:CYS:HB3	3:A:289:ILE:HD11	2.00	0.43
4:D:242:SER:HB3	4:D:245:MET:HE1	2.00	0.43

Continued on next page...

CONFIDENTIAL VALIDATION REPORT

Continued from previous page...

Atom-1	Atom-2	Interatomic distance (Å)	Clash overlap (Å)
1:E:21:VAL:HG12	1:E:35:LEU:HD23	2.00	0.43
1:E:127:GLU:HG3	1:E:135:TRP:CZ3	2.54	0.43
2:B:66:HIS:CD2	2:B:92:ASP:HB3	2.53	0.43
2:B:104:ILE:HD11	2:B:156:PHE:HE1	1.83	0.43
2:B:216:TRP:HZ3	2:B:225:PHE:CE2	2.36	0.43
3:A:203:THR:HG21	4:D:44:HIS:HB2	2.00	0.43
4:D:203:THR:HB	4:D:209:CYS:SG	2.58	0.43
3:A:155:SER:O	3:A:303:THR:HG22	2.18	0.43
1:E:29:MET:SD	5:C:590:SER:HB2	2.58	0.42
3:A:215:LYS:NZ	3:A:240:TYR:HE2	2.16	0.42
2:B:248:HIS:O	2:B:249:GLN:HG2	2.19	0.42
4:D:89:VAL:HG23	4:D:119:SER:O	2.20	0.42
2:B:170:PHE:O	2:B:244:ILE:HG13	2.20	0.42
2:B:250:VAL:HG22	2:B:267:PHE:CE2	2.55	0.42
2:B:295:ILE:HD12	5:C:562:VAL:CG1	2.50	0.42
4:D:99:ASP:O	4:D:107:ASP:HA	2.19	0.42
3:A:178:LEU:HG	3:A:180:LEU:HB3	2.02	0.42
1:E:53:ARG:NH2	2:B:130:ILE:HD11	2.35	0.42
1:E:45:ILE:O	1:E:45:ILE:HG13	2.19	0.42
4:D:117:LYS:HE3	4:D:158:TRP:CE2	2.55	0.42
2:B:92:ASP:OD1	2:B:125:HIS:HD2	2.03	0.42
2:B:173:HIS:CE1	2:B:248:HIS:HB2	2.55	0.42
1:E:45:ILE:HG22	1:E:84:LEU:HB2	2.02	0.42
2:B:43:ARG:HH12	2:B:154:ASP:HB3	1.85	0.42
3:A:132:MET:HE3	3:A:132:MET:HB3	1.94	0.41
4:D:67:VAL:HA	4:D:80:TYR:O	2.20	0.41
3:A:148:THR:HG22	5:C:603:LEU:HD12	2.02	0.41
5:C:592:PHE:O	5:C:596:ILE:HD12	2.20	0.41
1:E:21:VAL:HG11	1:E:84:LEU:HD13	2.01	0.41
1:E:140:LYS:HE2	1:E:141:TYR:CE2	2.55	0.41
2:B:60:LYS:O	2:B:87:TYR:HA	2.20	0.41
2:B:66:HIS:H	2:B:93:TYR:HA	1.86	0.41
2:B:66:HIS:NE2	2:B:92:ASP:HB3	2.36	0.41
2:B:164:ILE:HG12	2:B:170:PHE:HD1	1.86	0.41
2:B:233:ALA:O	2:B:236:LEU:HG	2.21	0.41
3:A:313:MET:HB2	3:A:329:ILE:HD13	2.03	0.41
3:A:123:MET:CE	3:A:129:VAL:HG11	2.50	0.41
3:A:335:ARG:HA	3:A:338:SER:HB3	2.03	0.40
5:C:596:ILE:HD12	5:C:596:ILE:H	1.85	0.40
2:B:13:ILE:HD11	2:B:109:ALA:HB1	2.03	0.40
3:A:162:ASN:HD22	3:A:278:THR:HA	1.86	0.40

Continued on next page...

CONFIDENTIAL VALIDATION REPORT

Continued from previous page...

Atom-1	Atom-2	Interatomic distance (Å)	Clash overlap (Å)
3:A:369:ILE:HD12	3:A:372:ARG:HE	1.86	0.40
2:B:219:ASN:HD22	2:B:225:PHE:C	2.20	0.40

There are no symmetry-related clashes.

5.3 Torsion angles (i)

5.3.1 Protein backbone (i)

In the following table, the Percentiles column shows the percent Ramachandran outliers of the chain as a percentile score with respect to all PDB entries followed by that with respect to all EM entries.

The Analysed column shows the number of residues for which the backbone conformation was analysed, and the total number of residues.

Mol	Chain	Analysed	Favoured	Allowed	Outliers	Percentiles	
1	E	175/186 (94%)	169 (97%)	6 (3%)	0	100	100
2	B	291/294 (99%)	272 (94%)	19 (6%)	0	100	100
3	A	368/375 (98%)	360 (98%)	8 (2%)	0	100	100
4	D	255/260 (98%)	249 (98%)	6 (2%)	0	100	100
5	C	57/444 (13%)	52 (91%)	5 (9%)	0	100	100
All	All	1146/1559 (74%)	1102 (96%)	44 (4%)	0	100	100

There are no Ramachandran outliers to report.

5.3.2 Protein sidechains (i)

In the following table, the Percentiles column shows the percent sidechain outliers of the chain as a percentile score with respect to all PDB entries followed by that with respect to all EM entries.

The Analysed column shows the number of residues for which the sidechain conformation was analysed, and the total number of residues.

Mol	Chain	Analysed	Rotameric	Outliers	Percentiles	
1	E	141/170 (83%)	141 (100%)	0	100	100
2	B	246/258 (95%)	245 (100%)	1 (0%)	91	94

Continued on next page...

CONFIDENTIAL VALIDATION REPORT

Continued from previous page...

Mol	Chain	Analysed	Rotameric	Outliers	Percentiles	
3	A	311/318 (98%)	311 (100%)	0	100	100
4	D	226/229 (99%)	226 (100%)	0	100	100
5	C	38/354 (11%)	38 (100%)	0	100	100
All	All	962/1329 (72%)	961 (100%)	1 (0%)	93	96

All (1) residues with a non-rotameric sidechain are listed below:

Mol	Chain	Res	Type
2	B	191	ARG

Sometimes sidechains can be flipped to improve hydrogen bonding and reduce clashes. There are no such sidechains identified.

5.3.3 RNA ⓘ

There are no RNA molecules in this entry.

5.4 Non-standard residues in protein, DNA, RNA chains ⓘ

1 non-standard protein/DNA/RNA residue is modelled in this entry.

In the following table, the Counts columns list the number of bonds (or angles) for which Mogul statistics could be retrieved, the number of bonds (or angles) that are observed in the model and the number of bonds (or angles) that are defined in the Chemical Component Dictionary. The Link column lists molecule types, if any, to which the group is linked. The Z score for a bond length (or angle) is the number of standard deviations the observed value is removed from the expected value. A bond length (or angle) with $|Z| > 2$ is considered an outlier worth inspection. RMSZ is the root-mean-square of all Z scores of the bond lengths (or angles).

Mol	Type	Chain	Res	Link	Bond lengths			Bond angles		
					Counts	RMSZ	$\#\|Z\| > 2$	Counts	RMSZ	$\#\|Z\| > 2$
1	SEP	E	51	1	8,9,10	1.47	1 (12%)	8,12,14	1.90	2 (25%)

In the following table, the Chirals column lists the number of chiral outliers, the number of chiral centers analysed, the number of these observed in the model and the number defined in the Chemical Component Dictionary. Similar counts are reported in the Torsion and Rings columns. '-' means no outliers of that kind were identified.

Mol	Type	Chain	Res	Link	Chirals	Torsions	Rings
1	SEP	E	51	1	-	0/5/8/10	-

CONFIDENTIAL VALIDATION REPORT

All (1) bond length outliers are listed below:

Mol	Chain	Res	Type	Atoms	Z	Observed(Å)	Ideal(Å)
1	E	51	SEP	P-O1P	3.28	1.61	1.50

All (2) bond angle outliers are listed below:

Mol	Chain	Res	Type	Atoms	Z	Observed(^{o})	Ideal(^{o})
1	E	51	SEP	P-OG-CB	-3.69	108.12	118.30
1	E	51	SEP	OG-CB-CA	3.30	111.36	108.14

There are no chirality outliers.

There are no torsion outliers.

There are no ring outliers.

1 monomer is involved in 2 short contacts:

Mol	Chain	Res	Type	Clashes	Symm-Clashes
1	E	51	SEP	2	0

5.5 Carbohydrates (i)

2 monosaccharides are modelled in this entry.

In the following table, the Counts columns list the number of bonds (or angles) for which Mogul statistics could be retrieved, the number of bonds (or angles) that are observed in the model and the number of bonds (or angles) that are defined in the Chemical Component Dictionary. The Link column lists molecule types, if any, to which the group is linked. The Z score for a bond length (or angle) is the number of standard deviations the observed value is removed from the expected value. A bond length (or angle) with $|Z| > 2$ is considered an outlier worth inspection. RMSZ is the root-mean-square of all Z scores of the bond lengths (or angles).

Mol	Type	Chain	Res	Link	Bond lengths			Bond angles		
					Counts	RMSZ	$\#\lvert Z\rvert > 2$	Counts	RMSZ	$\#\lvert Z\rvert > 2$
6	NAG	F	1	4,6	14,14,15	0.57	0	17,19,21	1.19	1 (5%)
6	NAG	F	2	6	14,14,15	0.90	1 (7%)	17,19,21	0.87	1 (5%)

In the following table, the Chirals column lists the number of chiral outliers, the number of chiral centers analysed, the number of these observed in the model and the number defined in the Chemical Component Dictionary. Similar counts are reported in the Torsion and Rings columns. '-' means no outliers of that kind were identified.

CONFIDENTIAL VALIDATION REPORT

Mol	Type	Chain	Res	Link	Chirals	Torsions	Rings
6	NAG	F	1	4,6	-	2/6/23/26	0/1/1/1
6	NAG	F	2	6	-	2/6/23/26	0/1/1/1

All (1) bond length outliers are listed below:

Mol	Chain	Res	Type	Atoms	Z	Observed(Å)	Ideal(Å)
6	F	2	NAG	C1-C2	2.66	1.56	1.52

All (2) bond angle outliers are listed below:

Mol	Chain	Res	Type	Atoms	Z	Observed(^{o})	Ideal(^{o})
6	F	1	NAG	C1-O5-C5	4.58	118.39	112.19
6	F	2	NAG	C1-O5-C5	3.17	116.48	112.19

There are no chirality outliers.

All (4) torsion outliers are listed below:

Mol	Chain	Res	Type	Atoms
6	F	1	NAG	O5-C5-C6-O6
6	F	2	NAG	O5-C5-C6-O6
6	F	1	NAG	C4-C5-C6-O6
6	F	2	NAG	C4-C5-C6-O6

There are no ring outliers.

1 monomer is involved in 1 short contact:

Mol	Chain	Res	Type	Clashes	Symm-Clashes
6	F	2	NAG	1	0

The following is a two-dimensional graphical depiction of Mogul quality analysis of bond lengths, bond angles, torsion angles, and ring geometry for oligosaccharide.

CONFIDENTIAL VALIDATION REPORT

Oligosaccharide Chain F

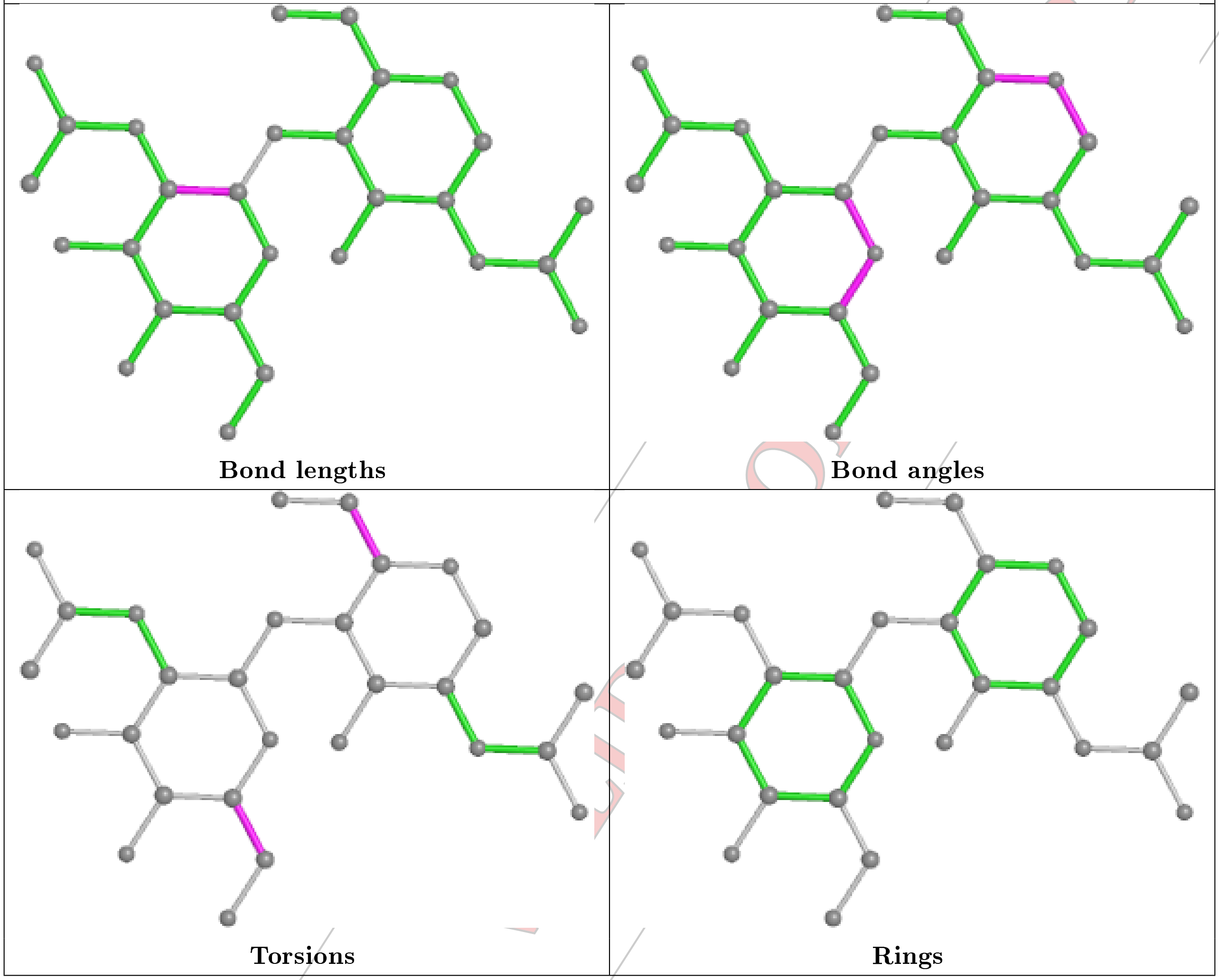

5.6 Ligand geometry (i)

Of 2 ligands modelled in this entry, 1 is monoatomic - leaving 1 for Mogul analysis.

In the following table, the Counts columns list the number of bonds (or angles) for which Mogul statistics could be retrieved, the number of bonds (or angles) that are observed in the model and the number of bonds (or angles) that are defined in the Chemical Component Dictionary. The Link column lists molecule types, if any, to which the group is linked. The Z score for a bond length (or angle) is the number of standard deviations the observed value is removed from the expected value. A bond length (or angle) with $|Z| > 2$ is considered an outlier worth inspection. RMSZ is the root-mean-square of all Z scores of the bond lengths (or angles).

Mol	Type	Chain	Res	Link	Bond lengths			Bond angles		
					Counts	RMSZ	$\#\|Z\| > 2$	Counts	RMSZ	$\#\|Z\| > 2$
8	ATP	A	401	-	26,33,33	0.93	1 (3%)	31,52,52	1.68	6 (19%)

In the following table, the Chirals column lists the number of chiral outliers, the number of chiral

CONFIDENTIAL REPORT

centers analysed, the number of these observed in the model and the number defined in the Chemical Component Dictionary. Similar counts are reported in the Torsion and Rings columns. '-' means no outliers of that kind were identified.

Mol	Type	Chain	Res	Link	Chirals	Torsions	Rings
8	ATP	A	401	-	-	2/18/38/38	0/3/3/3

All (1) bond length outliers are listed below:

Mol	Chain	Res	Type	Atoms	Z	Observed(Å)	Ideal(Å)
8	A	401	ATP	C5-C4	2.07	1.46	1.40

All (6) bond angle outliers are listed below:

Mol	Chain	Res	Type	Atoms	Z	Observed(^{o})	Ideal(^{o})
8	A	401	ATP	PA-O3A-PB	-5.09	115.35	132.83
8	A	401	ATP	PB-O3B-PG	-4.12	118.69	132.83
8	A	401	ATP	N3-C2-N1	-2.88	124.17	128.68
8	A	401	ATP	C4-C5-N7	-2.57	106.72	109.40
8	A	401	ATP	O2A-PA-O1A	2.08	122.50	112.24
8	A	401	ATP	O2B-PB-O1B	2.04	122.31	112.24

There are no chirality outliers.

All (2) torsion outliers are listed below:

Mol	Chain	Res	Type	Atoms
8	A	401	ATP	PG-O3B-PB-O1B
8	A	401	ATP	PG-O3B-PB-O2B

There are no ring outliers.

1 monomer is involved in 4 short contacts:

Mol	Chain	Res	Type	Clashes	Symm-Clashes
8	A	401	ATP	4	0

The following is a two-dimensional graphical depiction of Mogul quality analysis of bond lengths, bond angles, torsion angles, and ring geometry for all instances of the Ligand of Interest. In addition, ligands with molecular weight > 250 and outliers as shown on the validation Tables will also be included. For torsion angles, if less then 5% of the Mogul distribution of torsion angles is within 10 degrees of the torsion angle in question, then that torsion angle is considered an outlier. Any bond that is central to one or more torsion angles identified as an outlier by Mogul will be highlighted in the graph. For rings, the root-mean-square deviation (RMSD) between the ring in question and similar rings identified by Mogul is calculated over all ring torsion angles. If the

CONFIDENTIAL VALIDATION REPORT

average RMSD is greater than 60 degrees and the minimal RMSD between the ring in question and any Mogul-identified rings is also greater than 60 degrees, then that ring is considered an outlier. The outliers are highlighted in purple. The color gray indicates Mogul did not find sufficient equivalents in the CSD to analyse the geometry.

Ligand ATP A 401

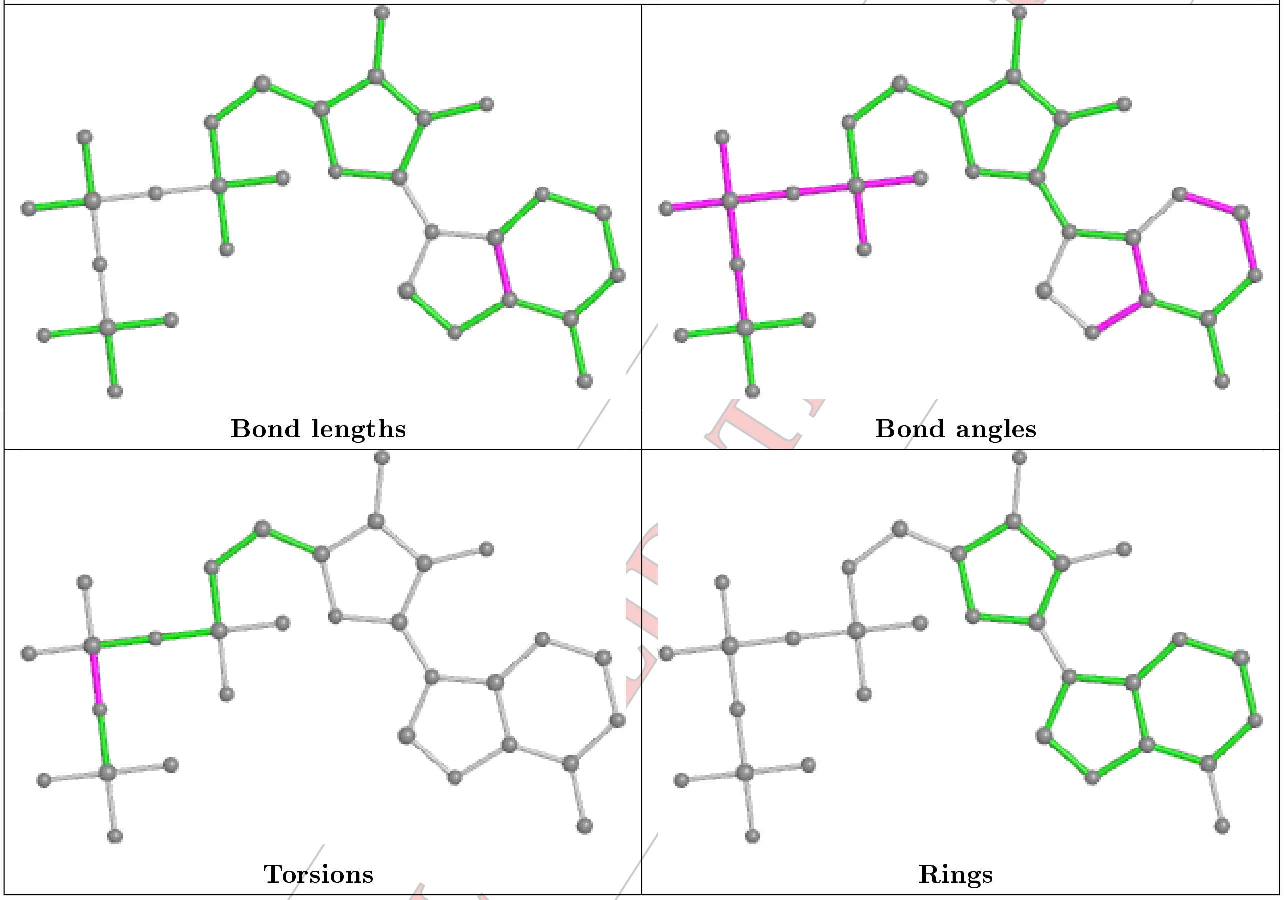

5.7 Other polymers (i)

There are no such residues in this entry.

5.8 Polymer linkage issues (i)

There are no chain breaks in this entry.

CONFIDENTIAL REPORT

6 Map visualisation (i)

This section contains visualisations of the EMDB entry EMD-12665. These allow visual inspection of the internal detail of the map and identification of artifacts.

Images derived from a raw map, generated by summing the deposited half-maps, are presented below the corresponding image components of the primary map to allow further visual inspection and comparison with those of the primary map.

6.1 Orthogonal projections (i)

6.1.1 Primary map

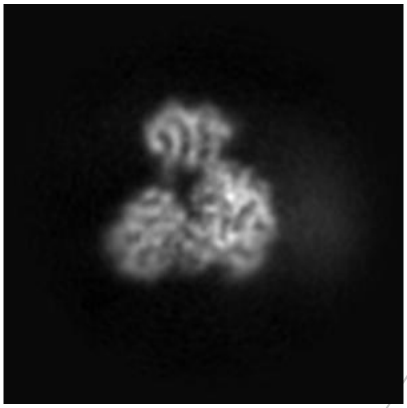

X

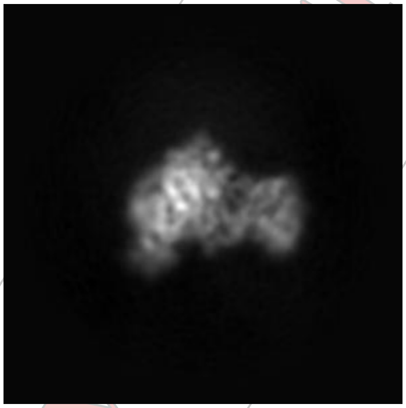

Y

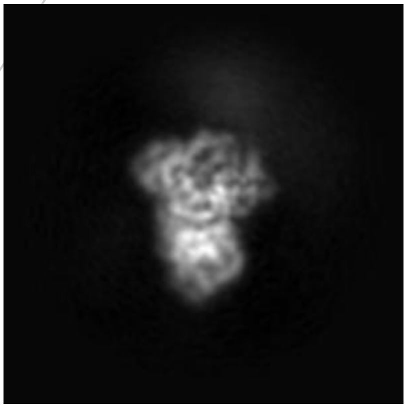

Z

6.1.2 Raw map

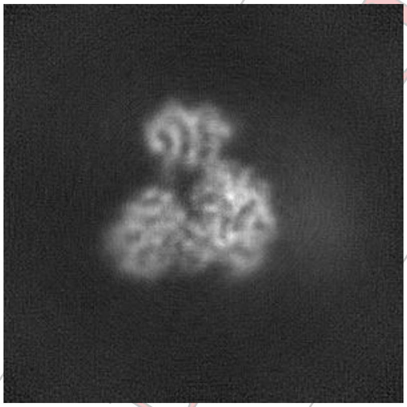

X

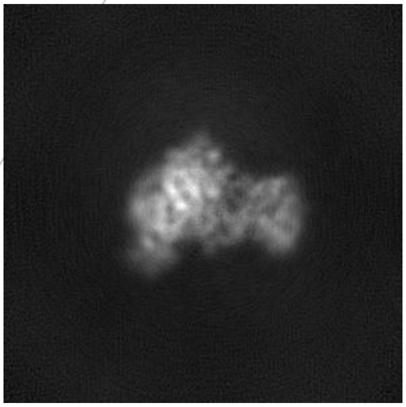

Y

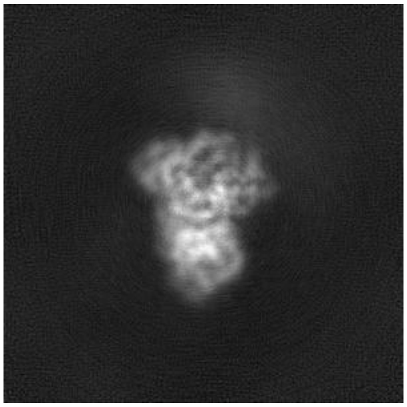

Z

The images above show the map projected in three orthogonal directions.

CONFIDENTIAL VALIDATION REPORT

6.2 Central slices (i)

6.2.1 Primary map

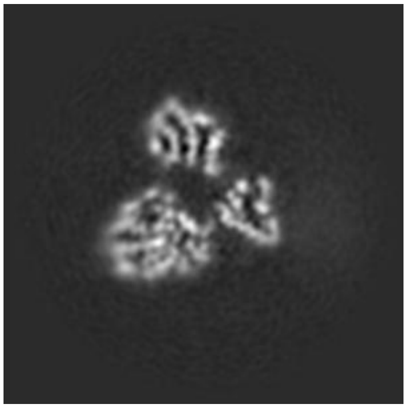

X Index: 170

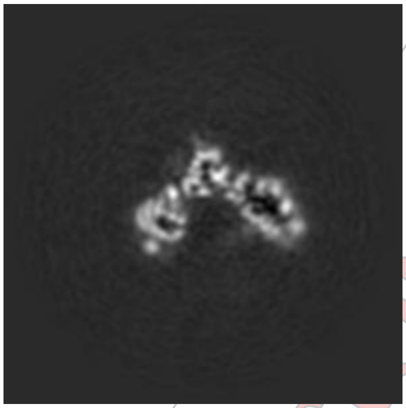

Y Index: 170

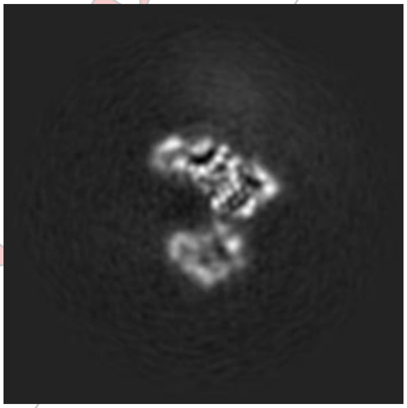

Z Index: 170

6.2.2 Raw map

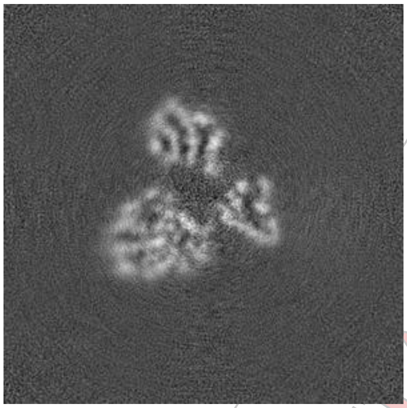

X Index: 170

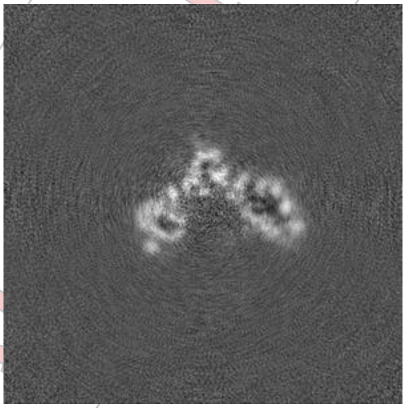

Y Index: 170

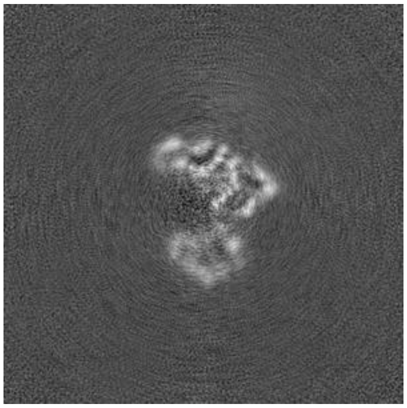

Z Index: 170

The images above show central slices of the map in three orthogonal directions.

CONFIDENTIAL VALIDATION REPORT

6.3 Largest variance slices (i)

6.3.1 Primary map

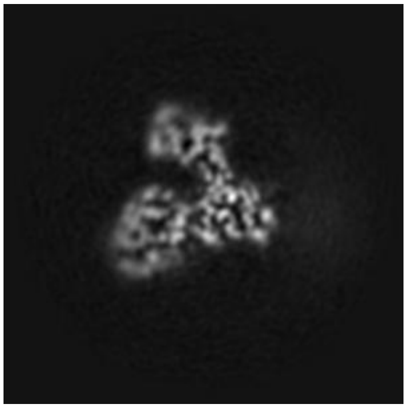

X Index: 181

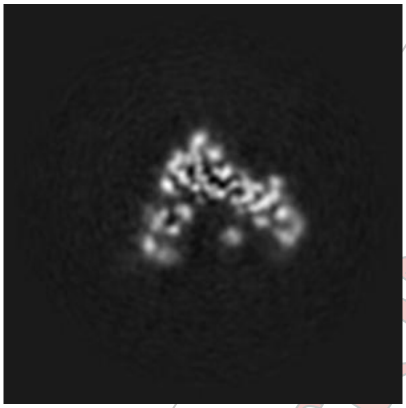

Y Index: 179

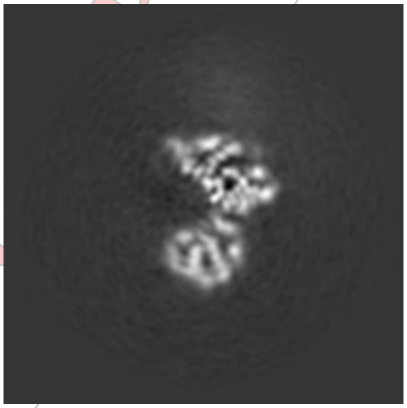

Z Index: 164

6.3.2 Raw map

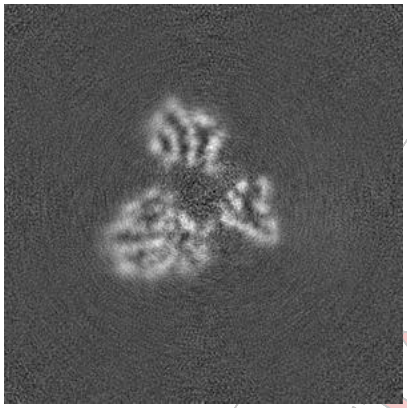

X Index: 169

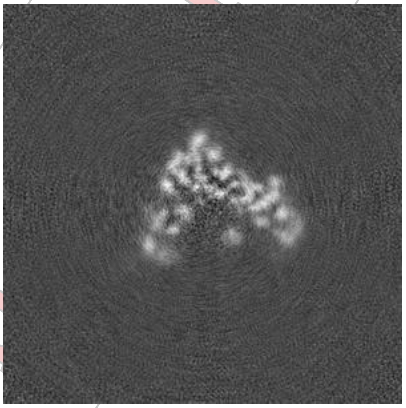

Y Index: 179

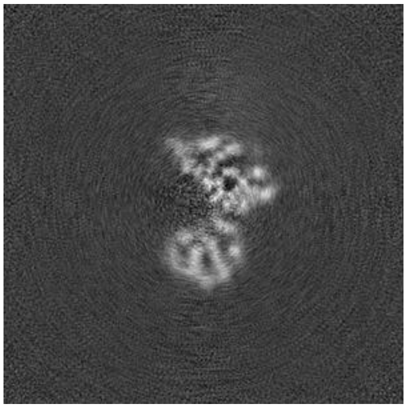

Z Index: 164

The images above show the largest variance slices of the map in three orthogonal directions.

CONFIDENTIAL VALIDATION REPORT

6.4 Orthogonal surface views

6.4.1 Primary map

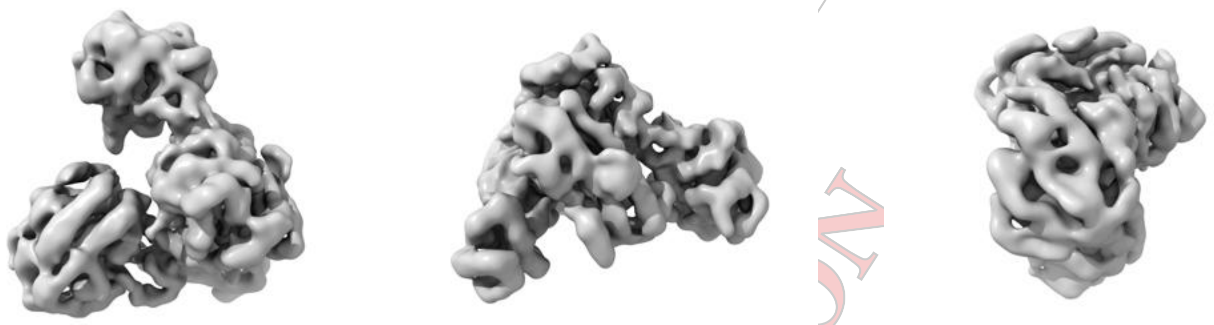

X Y Z

The images above show the 3D surface view of the map at the recommended contour level 0.05. These images, in conjunction with the slice images, may facilitate assessment of whether an appropriate contour level has been provided.

6.4.2 Raw map

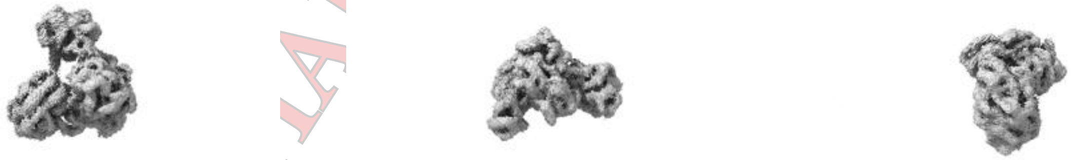

X Y Z

These images show the 3D surface of the raw map. The raw map's contour level was selected so that its surface encloses the same volume as the primary map does at its recommended contour level.

CONFIDENTIAL VALIDATION REPORT

6.5 Mask visualisation (i)

This section shows the 3D surface view of the primary map at 50% transparency overlaid with the specified mask at 0% transparency

A mask typically either:

- Encompasses the whole structure
- Separates out a domain, a functional unit, a monomer or an area of interest from a larger structure

6.5.1 D_1292114160_em-mask-volume_P1.map.V2 (i)

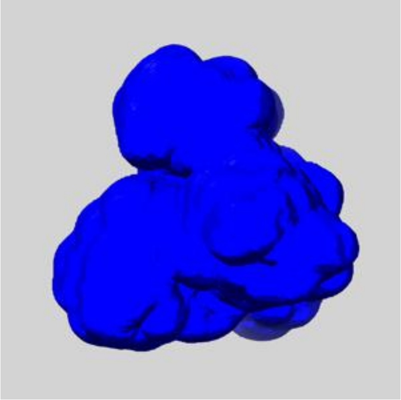

X

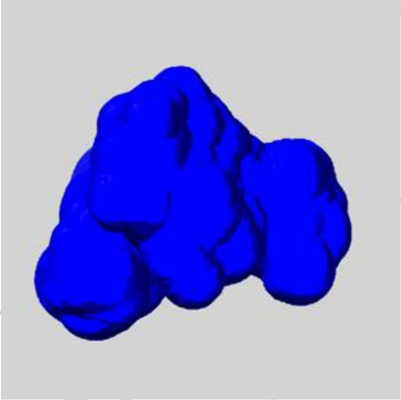

Y

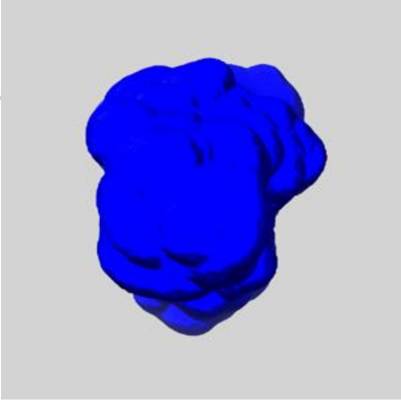

Z

CONFIDENTIAL VALIDATION REPORT

7 Map analysis (i)

This section contains the results of statistical analysis of the map.

7.1 Map-value distribution (i)

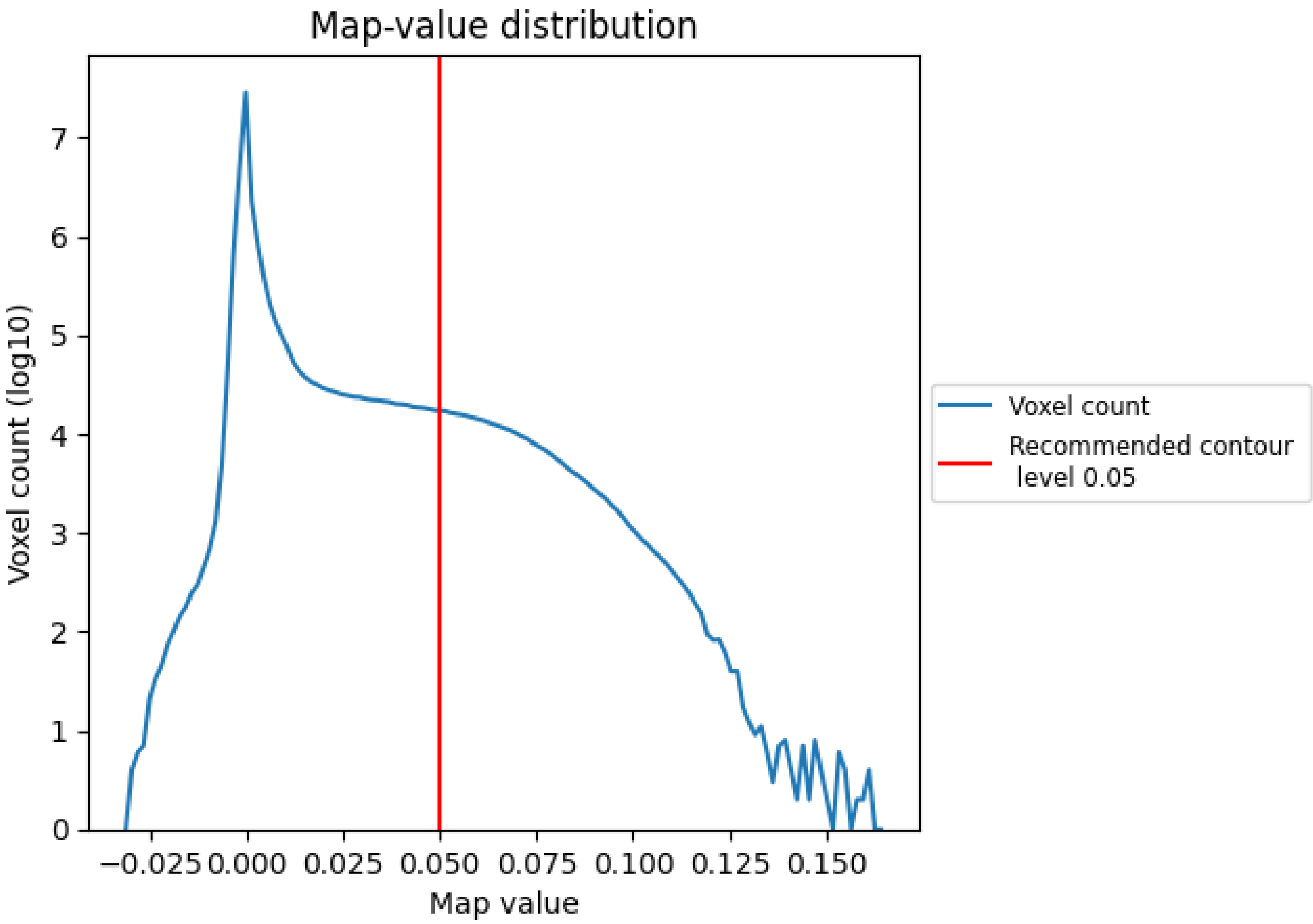

The map-value distribution is plotted in 128 intervals along the x-axis. The y-axis is logarithmic. A spike in this graph at zero usually indicates that the volume has been masked.

CONFIDENTIAL REPORT

7.2 Volume estimate ⓘ

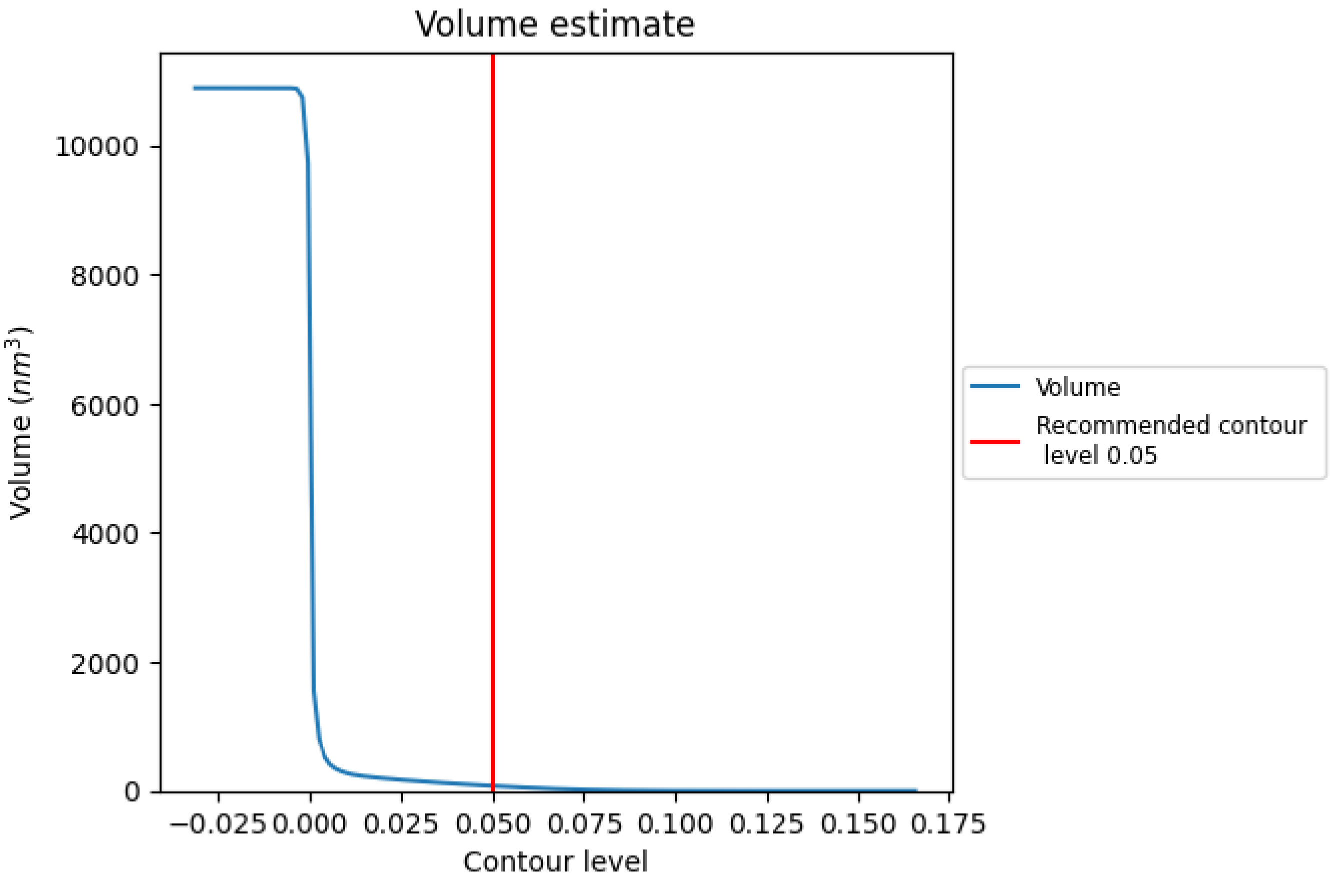

The volume at the recommended contour level is 79 nm^3; this corresponds to an approximate mass of 71 kDa.

The volume estimate graph shows how the enclosed volume varies with the contour level. The recommended contour level is shown as a vertical line and the intersection between the line and the curve gives the volume of the enclosed surface at the given level.

CONFIDENTIAL REPORT

7.3 Rotationally averaged power spectrum (i)

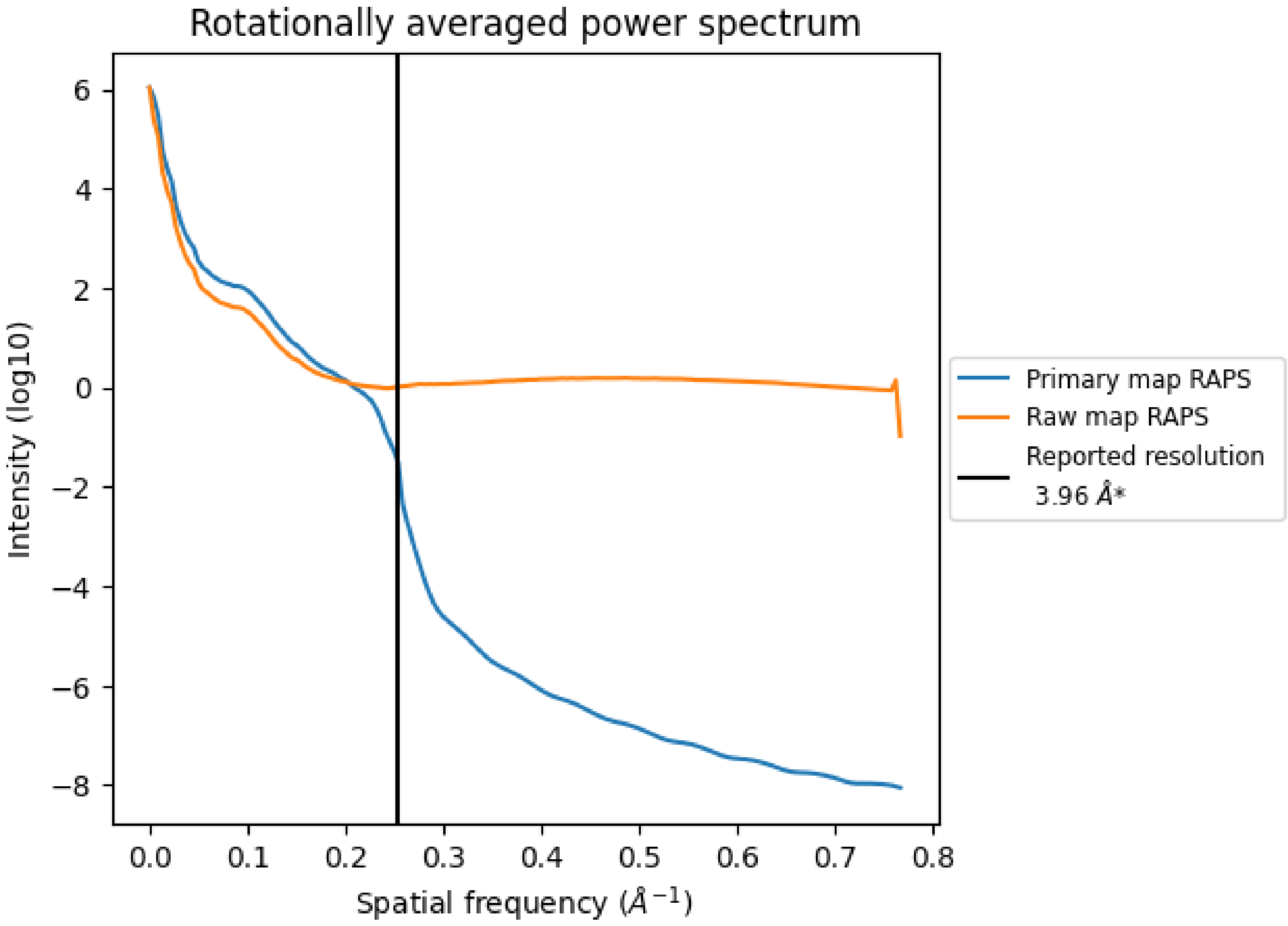

*Reported resolution corresponds to spatial frequency of 0.253 Å^{-1}

CONFIDENTIAL REPORT

8 Fourier-Shell correlation (i)

Fourier-Shell Correlation (FSC) is the most commonly used method to estimate the resolution of single-particle and subtomogram-averaged maps. The shape of the curve depends on the imposed symmetry, mask and whether or not the two 3D reconstructions used were processed from a common reference. The reported resolution is shown as a black line. A curve is displayed for the half-bit criterion in addition to lines showing the 0.143 gold standard cut-off and 0.5 cut-off.

8.1 FSC (i)

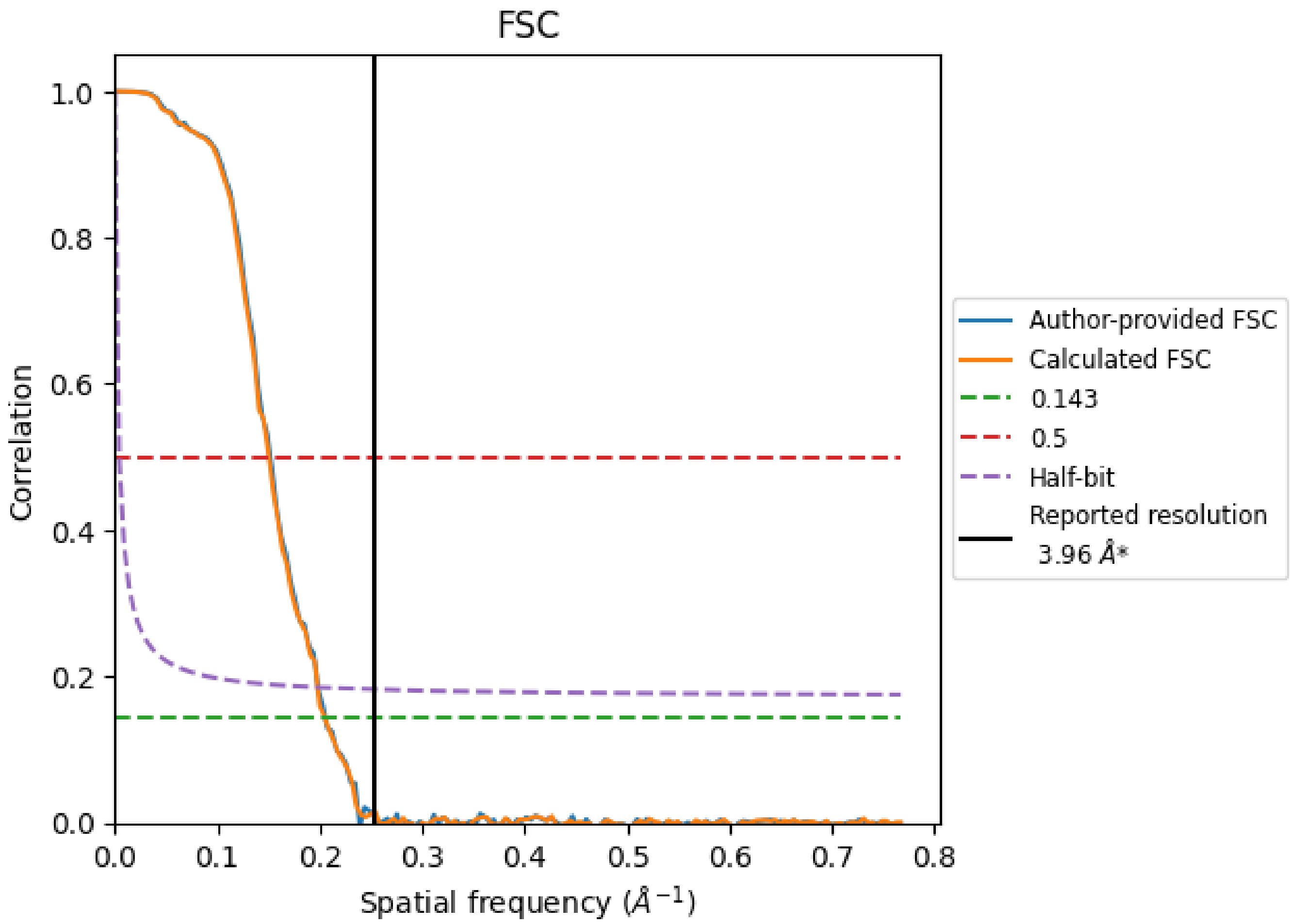

*Reported resolution corresponds to spatial frequency of 0.253 Å^{-1}

CONFIDENTIAL REPORT

8.2 Resolution estimates (i)

Resolution estimate (Å)	Estimation criterion (FSC cut-off)		
	0.143	0.5	Half-bit
Reported by author	3.96	-	-
Author-provided FSC curve	4.87	6.59	5.04
Calculated*	4.88	6.65	5.06

*Resolution estimate based on FSC curve calculated by comparison of deposited half-maps. The value from author-provided FSC intersecting FSC 0.143 CUT-OFF 4.87 differs from the reported value 3.96 by more than 10 %

The value from deposited half-maps intersecting FSC 0.143 CUT-OFF 4.88 differs from the reported value 3.96 by more than 10 %

CONFIDENTIAL VALIDATION REPORT

9 Map-model fit (i)

This section contains information regarding the fit between EMDB map EMD-12665 and PDB model 7NZM. Per-residue inclusion information can be found in section 3 on page 6.

9.1 Map-model overlay (i)

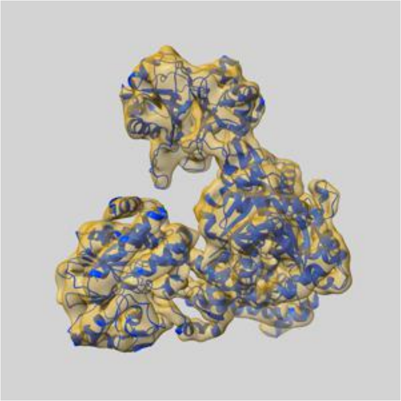

X

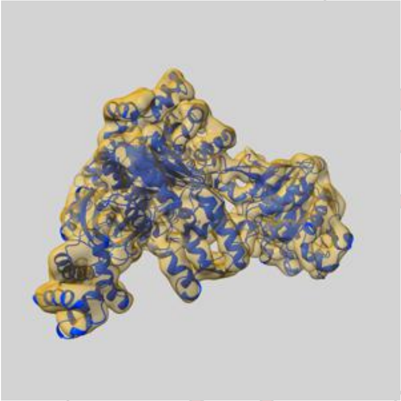

Y

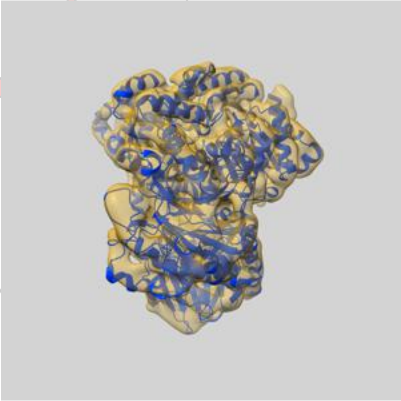

Z

The images above show the 3D surface view of the map at the recommended contour level 0.05 at 50% transparency in yellow overlaid with a ribbon representation of the model coloured in blue. These images allow for the visual assessment of the quality of fit between the atomic model and the map.

CONFIDENTIAL VALIDATION REPORT

9.2 Atom inclusion (i)

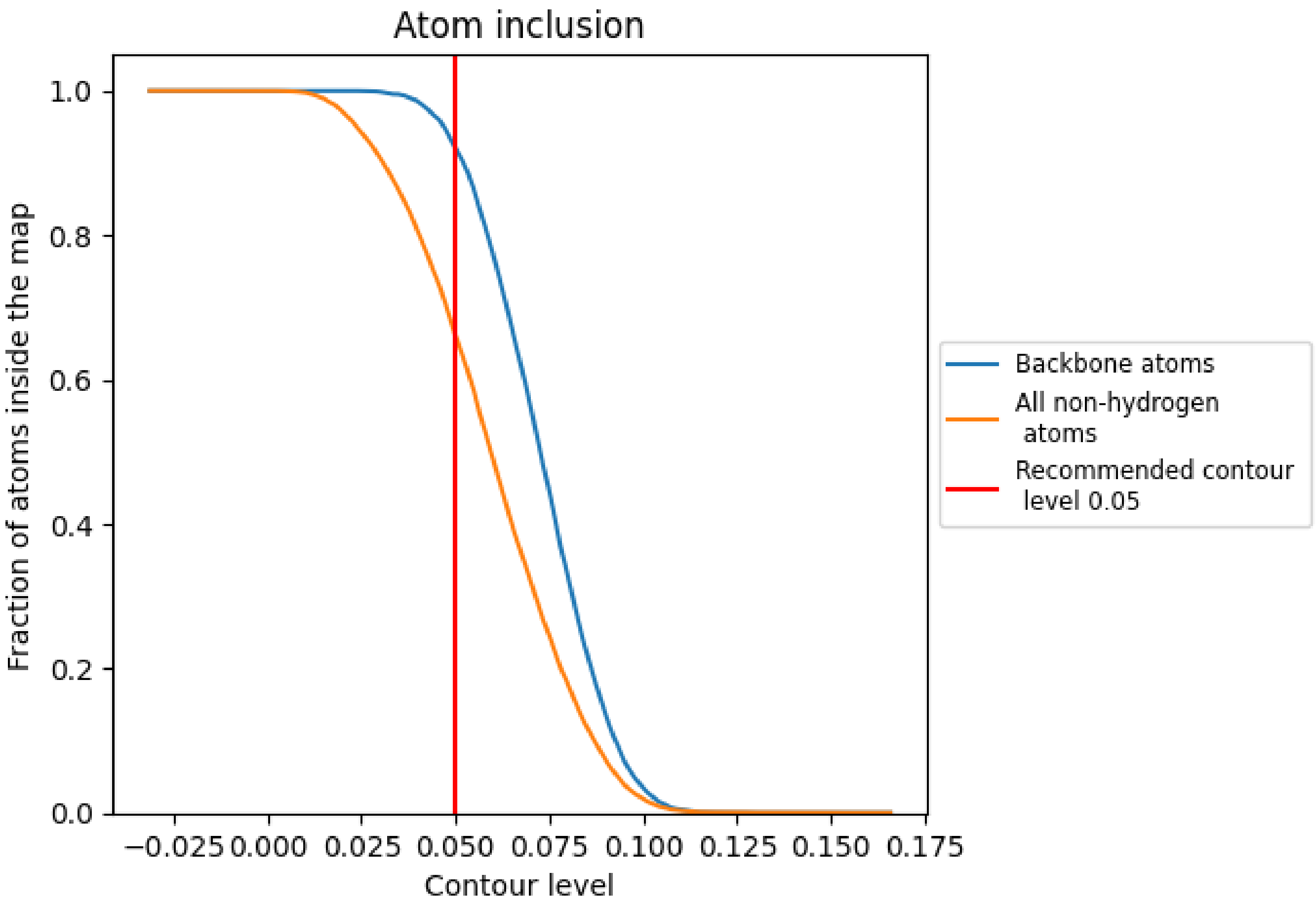

At the recommended contour level, 92% of all backbone atoms, 66% of all non-hydrogen atoms, are inside the map.

CONFIDENTIAL REPORT
