# Supplementary material for: Higher-order phosphatase–substrate contacts terminate the integrated stress response
Source: Nat Struct Mol Biol. 2021 Oct 8;28(10):835–46. doi: 10.1038/s41594-021-00666-7 (PMC8500838; doi:10.1038/s41594-021-00666-7)
Supplement: Supplementary file 2 — Reporting Summary [file 41594_2021_666_MOESM2_ESM.pdf]

nature research

Corresponding author(s): David Ron

Last updated by author(s): Aug 11, 2021

Reporting Summary

Nature Research wishes to improve the reproducibility of the work that we publish. This form provides structure for consistency and transparency in reporting. For further information on Nature Research policies, see our Editorial Policies and the Editorial Policy Checklist.

Statistics

For all statistical analyses, confirm that the following items are present in the figure legend, table legend, main text, or Methods section.

n/a	Confirmed	
☐	☒	The exact sample size (*n*) for each experimental group/condition, given as a discrete number and unit of measurement
☒	☐	A statement on whether measurements were taken from distinct samples or whether the same sample was measured repeatedly
☐	☒	The statistical test(s) used AND whether they are one- or two-sided<br>*Only common tests should be described solely by name; describe more complex techniques in the Methods section.*
☒	☐	A description of all covariates tested
☒	☐	A description of any assumptions or corrections, such as tests of normality and adjustment for multiple comparisons
☐	☒	A full description of the statistical parameters including central tendency (e.g. means) or other basic estimates (e.g. regression coefficient) AND variation (e.g. standard deviation) or associated estimates of uncertainty (e.g. confidence intervals)
☐	☒	For null hypothesis testing, the test statistic (e.g. *F*, *t*, *r*) with confidence intervals, effect sizes, degrees of freedom and *P* value noted<br>*Give P values as exact values whenever suitable.*
☒	☐	For Bayesian analysis, information on the choice of priors and Markov chain Monte Carlo settings
☒	☐	For hierarchical and complex designs, identification of the appropriate level for tests and full reporting of outcomes
☒	☐	Estimates of effect sizes (e.g. Cohen's *d*, Pearson's *r*), indicating how they were calculated

Our web collection on statistics for biologists contains articles on many of the points above.

Software and code

Policy information about availability of computer code

Data collection	The Cryo-EM data was collected using software EPU 2.<br>The X-ray crystal structure data was collected in Diamond Light Source (DLS) using DLS developed Generic Data Acquisition (GDA) software (v9.2).<br>The bio-layer interferometry was collected from Octet red 96 using the data requisition software in Octet.<br>The flow cytometry data was acquired from BD Flow cytometer using FACSDIVA (v8.0.1, BD Bioscience).<br>The fluorescence images were acquired with EVOS M5000 1.0.466.664.
Data analysis	The software for structure determination and illustration includes Phenix1.18.2-3874, ccp4i2-7.1.012, COOT-0.9.2, UCSF Chimera-1.14, WARP 1.0.6, CryoSPARC-v3.1, UCSF Pyem-v0.5 and Pymol 1.3. Prism 9 was used for statistics and presentation of biochemical and cell based experiments. FlowJo 8.0, NIH Fiji v1.0 and Excel2016 were used for flow cytometry data analysis.

For manuscripts utilizing custom algorithms or software that are central to the research but not yet described in published literature, software must be made available to editors and reviewers. We strongly encourage code deposition in a community repository (e.g. GitHub). See the Nature Research guidelines for submitting code & software for further information.

Data

Policy information about availability of data

All manuscripts must include a data availability statement. This statement should provide the following information, where applicable:

- Accession codes, unique identifiers, or web links for publicly available datasets
- A list of figures that have associated raw data
- A description of any restrictions on data availability

The atomic coordinates and structure factors of the crystal structure of DNase I/G-actin/PPP1R15A582-621 complex have been deposited to the PDB with accession code PDB 7NXV. Electron microscope density maps and atomic models of the pre-dephosphorylation complex have been deposited in the EMDB and PDB, respectively, with accession codes EMD-12665 and PDB 7NZM. Structures under PDB accession codes 4MOV, 2A42 and 1KL9 were used as initial models for refinement. Other structures used as alignment for illustration are available in PDB, including 3EKS, 3J8A, 2A19, 6I3M, 6K71, 7D45, 3JAP,1Q46, 6K72, 3CW2, 3JAP.

Field-specific reporting

Please select the one below that is the best fit for your research. If you are not sure, read the appropriate sections before making your selection.

- [x] Life sciences
- [ ] Behavioural & social sciences
- [ ] Ecological, evolutionary & environmental sciences

For a reference copy of the document with all sections, see nature.com/documents/nr-reporting-summary-flat.pdf

Life sciences study design

All studies must disclose on these points even when the disclosure is negative.

Sample size	Sample sizes for cell-based experiments are indicated in the relevant figures and the Flow Cytometry section. This number of cells is known to produce a signal-to-noise ratio (in the fluorescence) which is more than sufficient to observe any significant variations between different cell populations. Crystallography and Cryo-EM structures were processed from the best data set after extensive optimization of sample prep and data process.
Data exclusions	No data was excluded.
Replication	In general, at least 3 biological independent experimental replication were applied, which is commonly accepted to be efficient and practical to show significance and reproducibility. The exact replicate numbers are indicated when relevant.
Randomization	Cells were split randomly for different treatment as indicated in FACS experiments.
Blinding	No blinding experiments are included. An individual person conducted each experiment according to their expertise.

Reporting for specific materials, systems and methods

We require information from authors about some types of materials, experimental systems and methods used in many studies. Here, indicate whether each material, system or method listed is relevant to your study. If you are not sure if a list item applies to your research, read the appropriate section before selecting a response.

Materials & experimental systems

n/a	Involved in the study	
☒	☐	Antibodies
☐	☒	Eukaryotic cell lines
☒	☐	Palaeontology and archaeology
☒	☐	Animals and other organisms
☒	☐	Human research participants
☒	☐	Clinical data
☒	☐	Dual use research of concern

Methods

n/a	Involved in the study	
☒	☐	ChIP-seq
☐	☒	Flow cytometry
☒	☐	MRI-based neuroimaging

Eukaryotic cell lines

Policy information about cell lines

Cell line source(s)	CHO-K1 were sourced from ATCC (CCL-61) and then transduced with a CHOP::GFP reporter as previously reported in PMID:11381086.
Authentication	Cells and their derivatives were authenticated by PCR with species specific primers and CRISPR mutagenesis as reported previously (PMID 33220178 and 31749445).

Mycoplasma contamination	Negative tested.
Commonly misidentified lines (See ICLAC register)	None.

Flow Cytometry

Plots

Confirm that:

☒ The axis labels state the marker and fluorochrome used (e.g. CD4-FITC).

☒ The axis scales are clearly visible. Include numbers along axes only for bottom left plot of group (a 'group' is an analysis of identical markers).

☒ All plots are contour plots with outliers or pseudocolor plots.

☒ A numerical value for number of cells or percentage (with statistics) is provided.

Methodology

Sample preparation	Cultured cell line; samples were treated and then collected in PBS-EDTA.
Instrument	BD Flowcytometer Fortessas
Software	BD Flowcytometer FACSDIVA (v8.0.1, BD Bioscience).
Cell population abundance	Approximately 10,000 transfected cells for each sample.
Gating strategy	FSC/SSC was used to identify live singlet cells and then 10,000 transfected cells were gated using fluorescent proteins (BFP or mCherry) as indicated in the legends.

☒ Tick this box to confirm that a figure exemplifying the gating strategy is provided in the Supplementary Information.
