# Supplementary material for: Higher-order phosphatase–substrate contacts terminate the integrated stress response
Source: Nat Struct Mol Biol. 2021 Oct 8;28(10):835–46. doi: 10.1038/s41594-021-00666-7 (PMC8500838; doi:10.1038/s41594-021-00666-7)
Supplement: Source Data Fig. 2 — Unprocessed gels. [file 41594_2021_666_MOESM6_ESM.pdf]

Original gels for Fig. 2a

Phos-tag gel 1 (R15/PP1A 8nM)

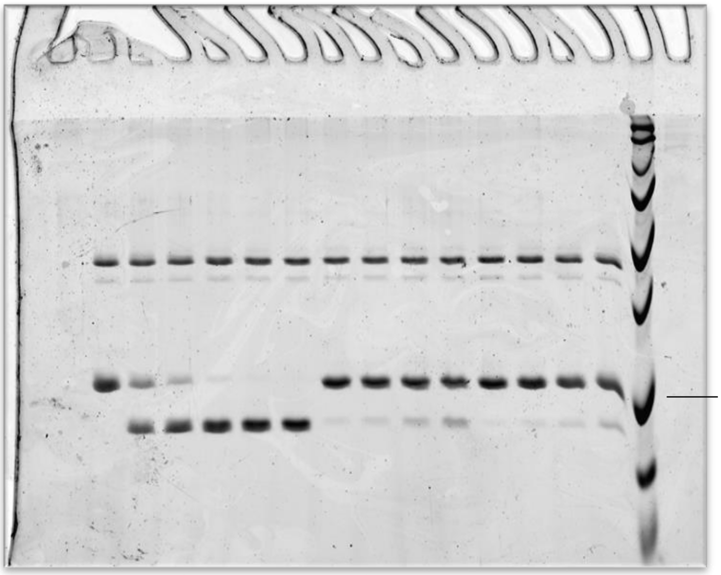

Phos-tag gel 2 (R15/PP1A 8nM)

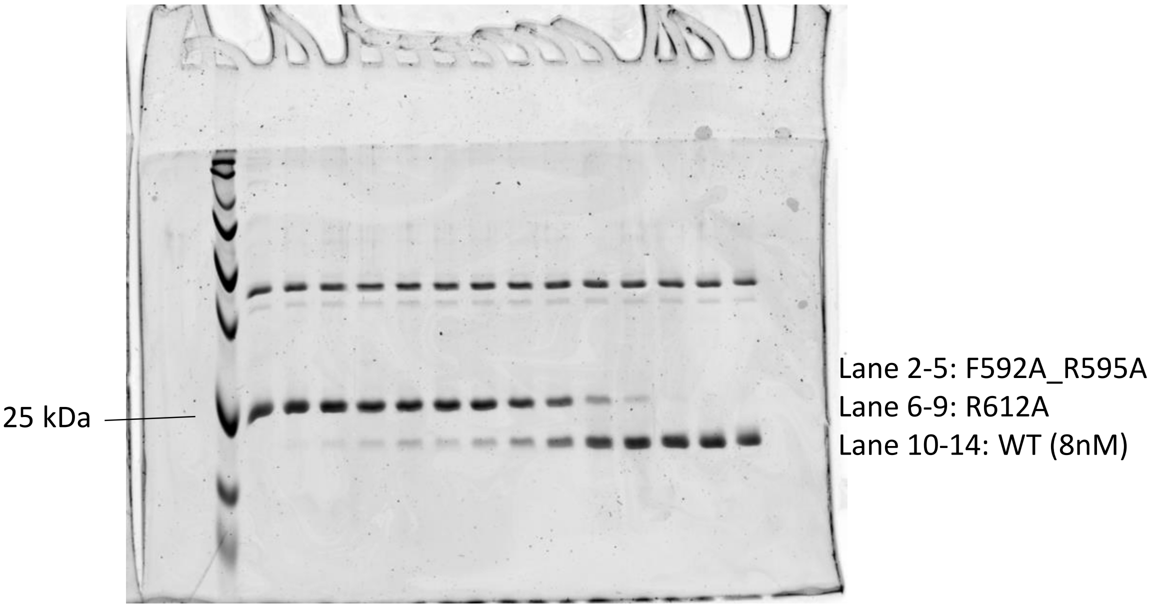

Phos-tag gel 3

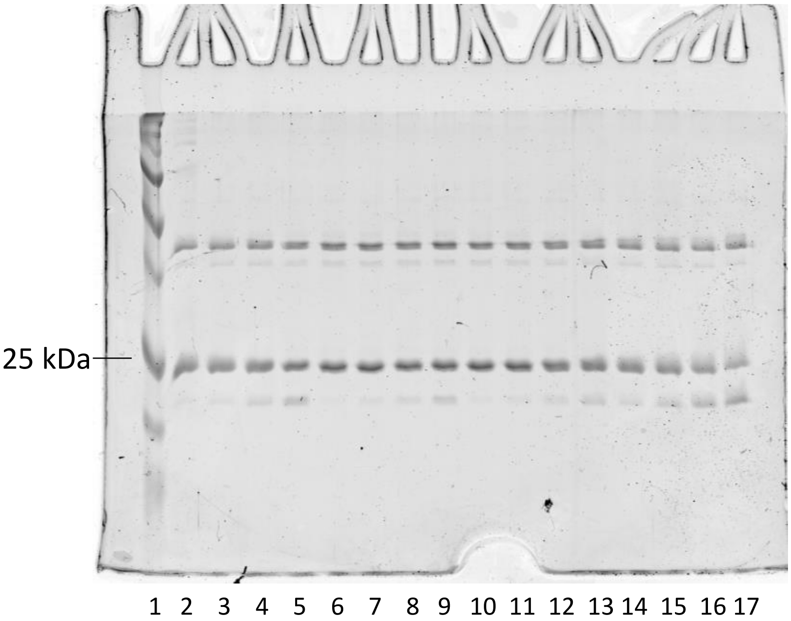

Lane 2-5: R595E
Lane 6-9: F592A_R595E
Lane 10-13: F592A_R595A
Lane 14-17: R612

Phos-tag gel 4

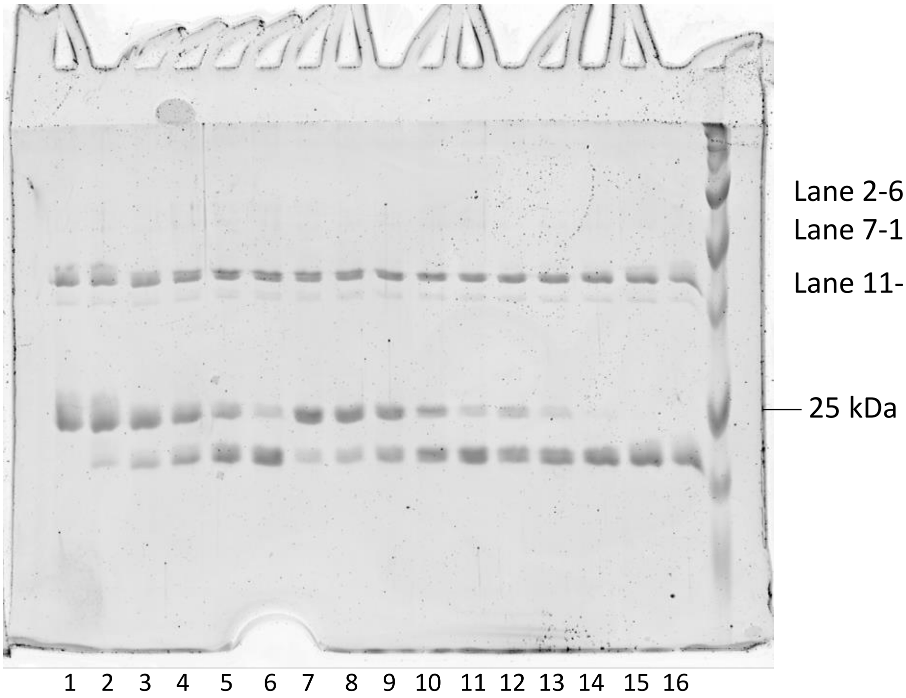

Lane 2-6: WT (2nM)
Lane 7-11: WT (2nM)
Lane 11-16: WT (8nM)

Phos-tag gel 5

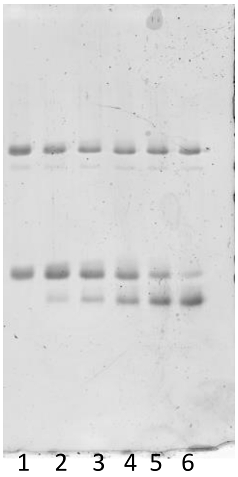

Lane 1-6: WT (2nM)
