# Supplementary material for: Higher-order phosphatase–substrate contacts terminate the integrated stress response
Source: Nat Struct Mol Biol. 2021 Oct 8;28(10):835–46. doi: 10.1038/s41594-021-00666-7 (PMC8500838; doi:10.1038/s41594-021-00666-7)
Supplement: Source Data Fig. 5 — Unprocessed gels. [file 41594_2021_666_MOESM9_ESM.pdf]

Original Phos-tag SDS PAGE for Fig. 5a

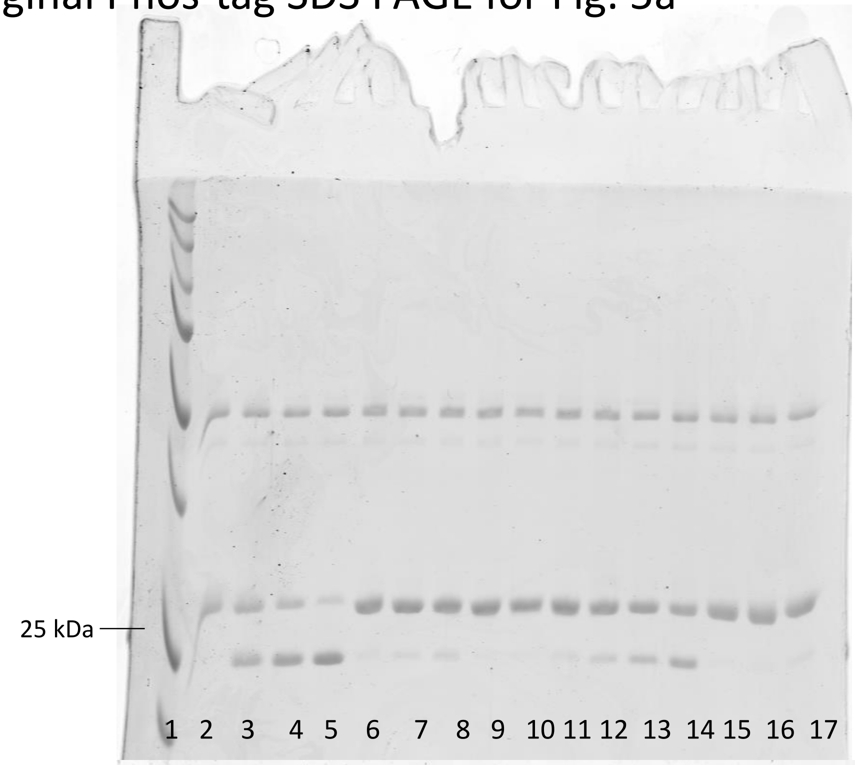

Full gel for Figure 6a top:

lane 3-5: WT
lane 6-8: W582A
lane 9-11: R591A
lane 12-14: R594A
lane 15-17: R591/594A

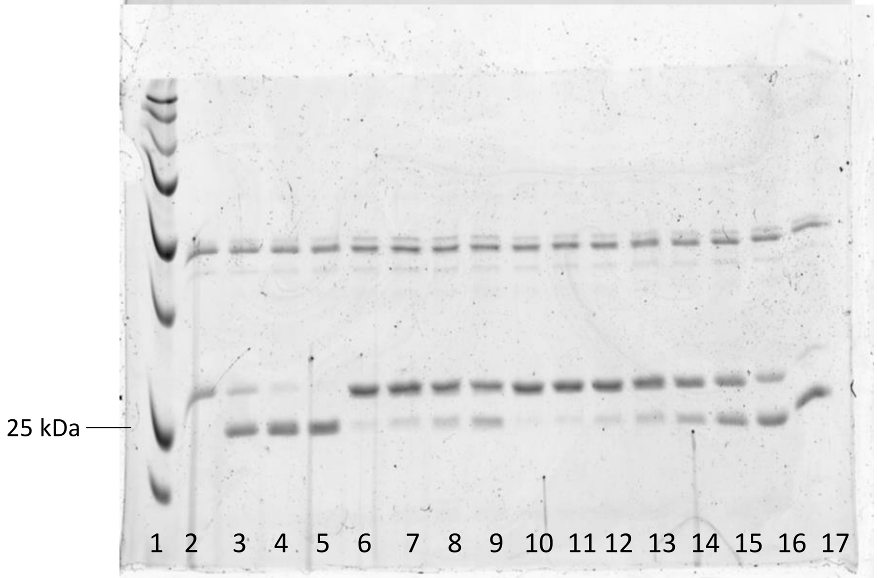

Full gel for Figure 6a bottom:

lane 3-5: WT
lane 6-9: W582A
lane 10-13: R591A
lane 14-17: R594A

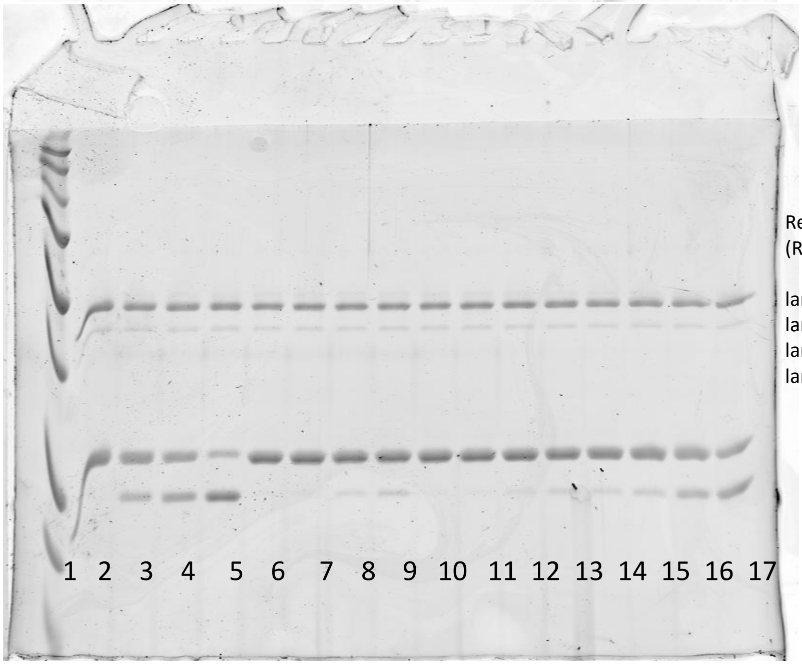

Repeat of Fig. 5a
(R15/PP1A: 2.7nM)

lane 3-5: WT (5min, 10min, 30min)
lane 6-9: W582A (5min, 10min, 30min, 60min)
lane 10-13: R591A (5min, 10min, 30min, 60min)
lane 14-17: R594A (5min, 10min, 30min, 60min)

Original Phos-tag SDS PAGE for Fig. 5b

50min

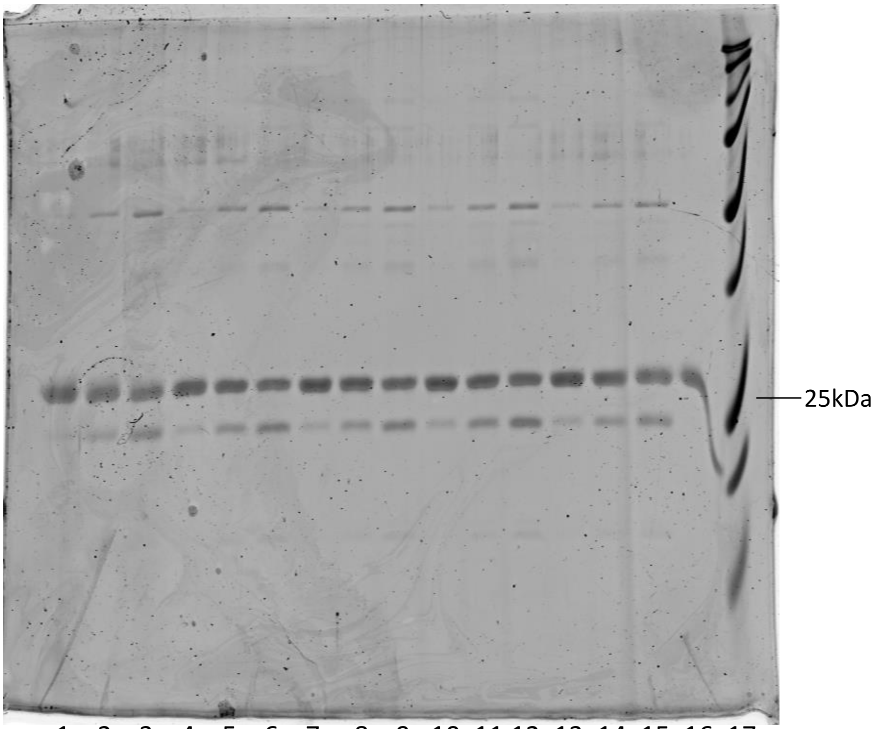

1 2 3 4 5 6 7 8 9 10 11 12 13 14 15 16 17

lane	R15/PP1 (nM)	R15A mutation
1	8	
2	32	
3	64	WT
4	8	
5	32	
6	64	W582A
7	8	
8	32	
9	64	R591A
10	8	
11	32	
12	64	R594A
13	8	
14	32	R591A;
15	64	R594A
16	NA	eIF2a-NTD-P
17	NA	MWM

25min

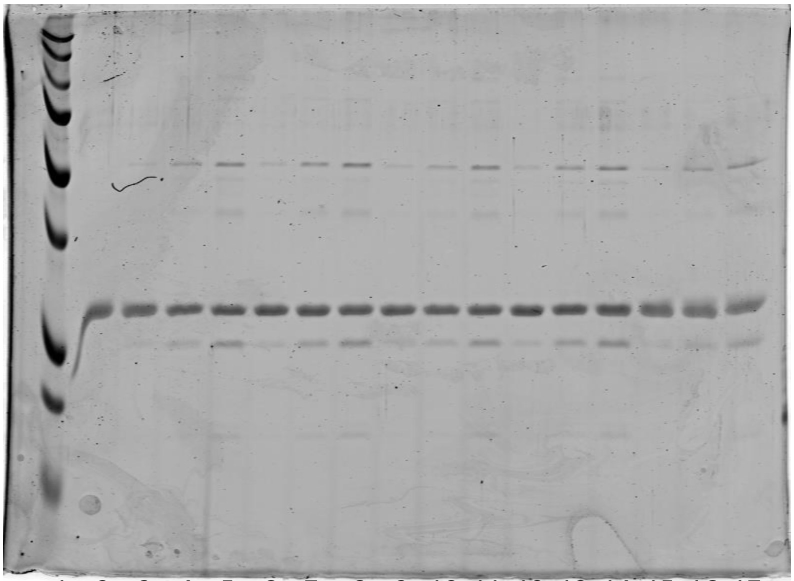

1 2 3 4 5 6 7 8 9 10 11 12 13 14 15 16 17

lane	R15/PP1 (nM)	R15A mutation
1	NA	MWM
2	NA	2a-P
3	8	
4	32	
5	64	WT
6	8	
7	32	
8	64	W582A
9	8	
10	32	
11	64	R591A
12	8	
13	32	
14	64	R594A
15	8	
16	32	
17	64	R591A; R594A
