# Supplementary material for: Higher-order phosphatase–substrate contacts terminate the integrated stress response
Source: Nat Struct Mol Biol. 2021 Oct 8;28(10):835–46. doi: 10.1038/s41594-021-00666-7 (PMC8500838; doi:10.1038/s41594-021-00666-7)
Supplement: Source Data Fig. 6 — Unprocessed gels. [file 41594_2021_666_MOESM11_ESM.pdf]

Original Phos-tag SDS PAGE for Fig. 6b

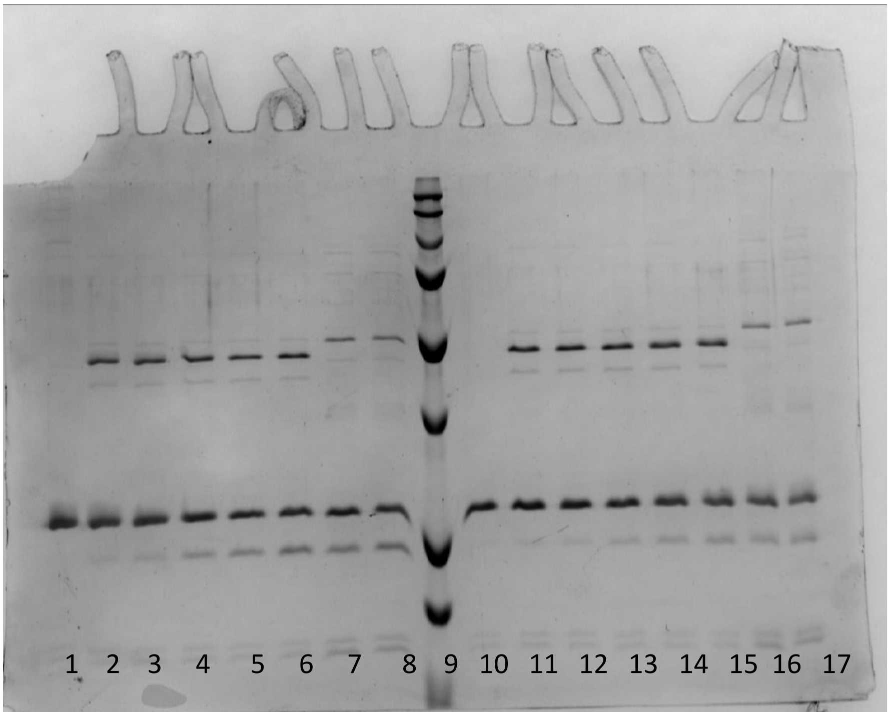

Full gel for Fig 6b top (Exp 6)
Lane 1-6: K79A(with actin)
lane 7-8: K79A (no actin)
lane 10-15: Y81A (with actin)
lane 16-17: Y81A (no actin)

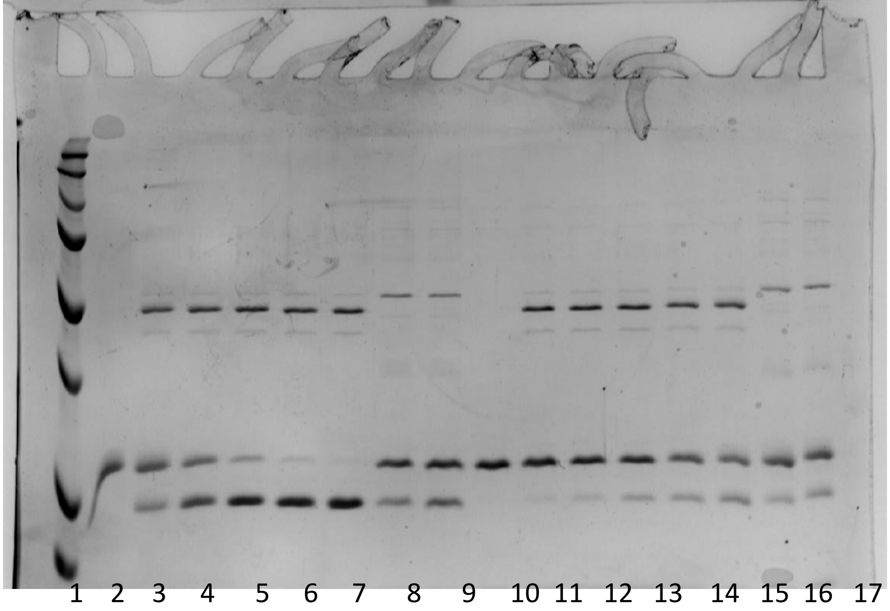

Full gel for Fig 6b top (Exp 6)
Lane 2-7: WT (with actin)
lane 8-9: WT (no actin)
lane 10-15 M44A (with actin)
lane 16-17: M44A (no actin)

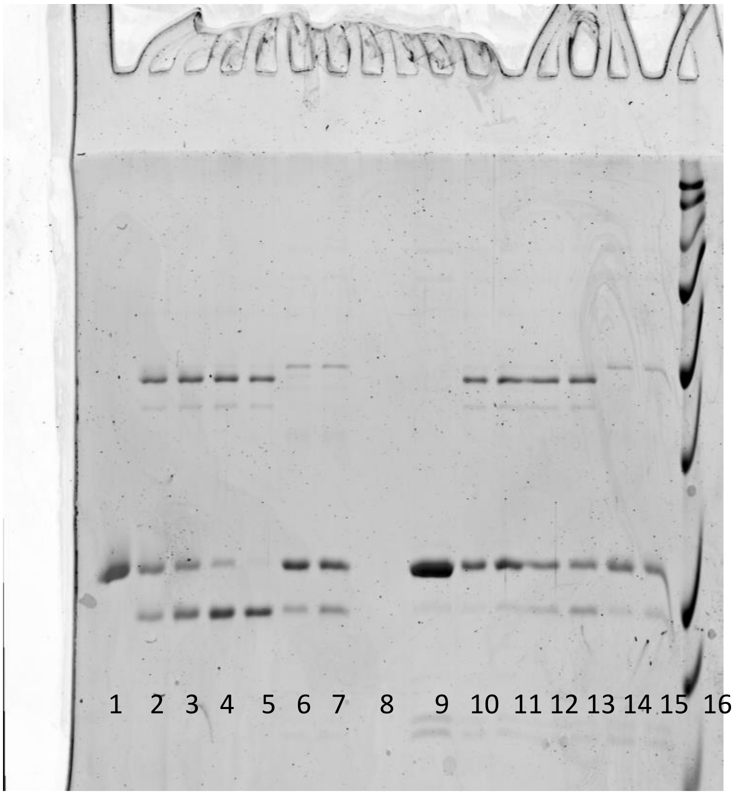

Repeated gel for Fig 6b top (Exp 5)
Lane 1-5: WT (with actin)
lane 6-7: WT (no actin)
lane 9-13 M44A (with actin)
lane 14-15: M44A (no actin)

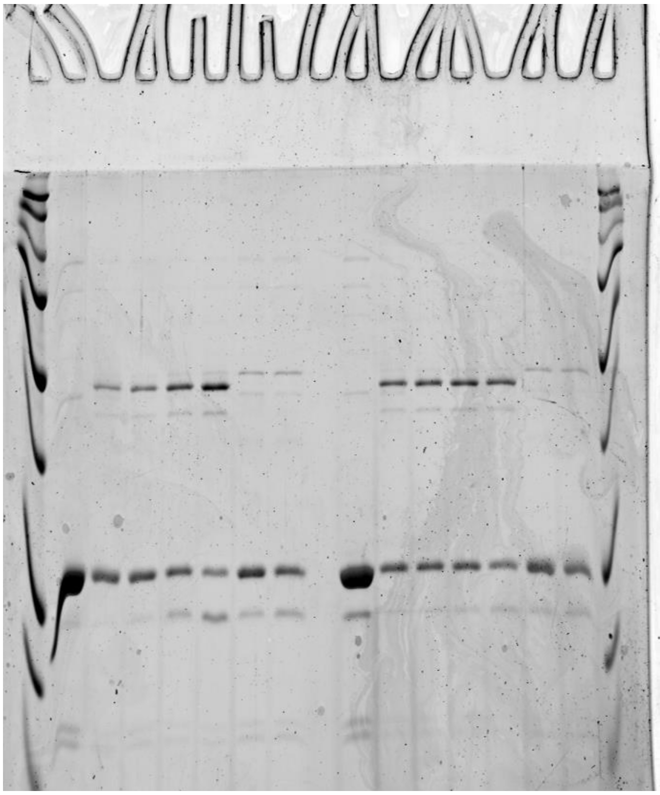

1 2 3 4 5 6 7 8 9 10 11 12 13 14 15 16

Repeated gel for Fig 6b top (Exp 5):
Lane 2-6: K79A(with actin)
lane 7-8: K79A (no actin)
lane 9-13: Y81A (with actin)
lane 14-15: Y81A (no actin)

Original Phos-tag SDS PAGE for Fig. 6c

EXP 3

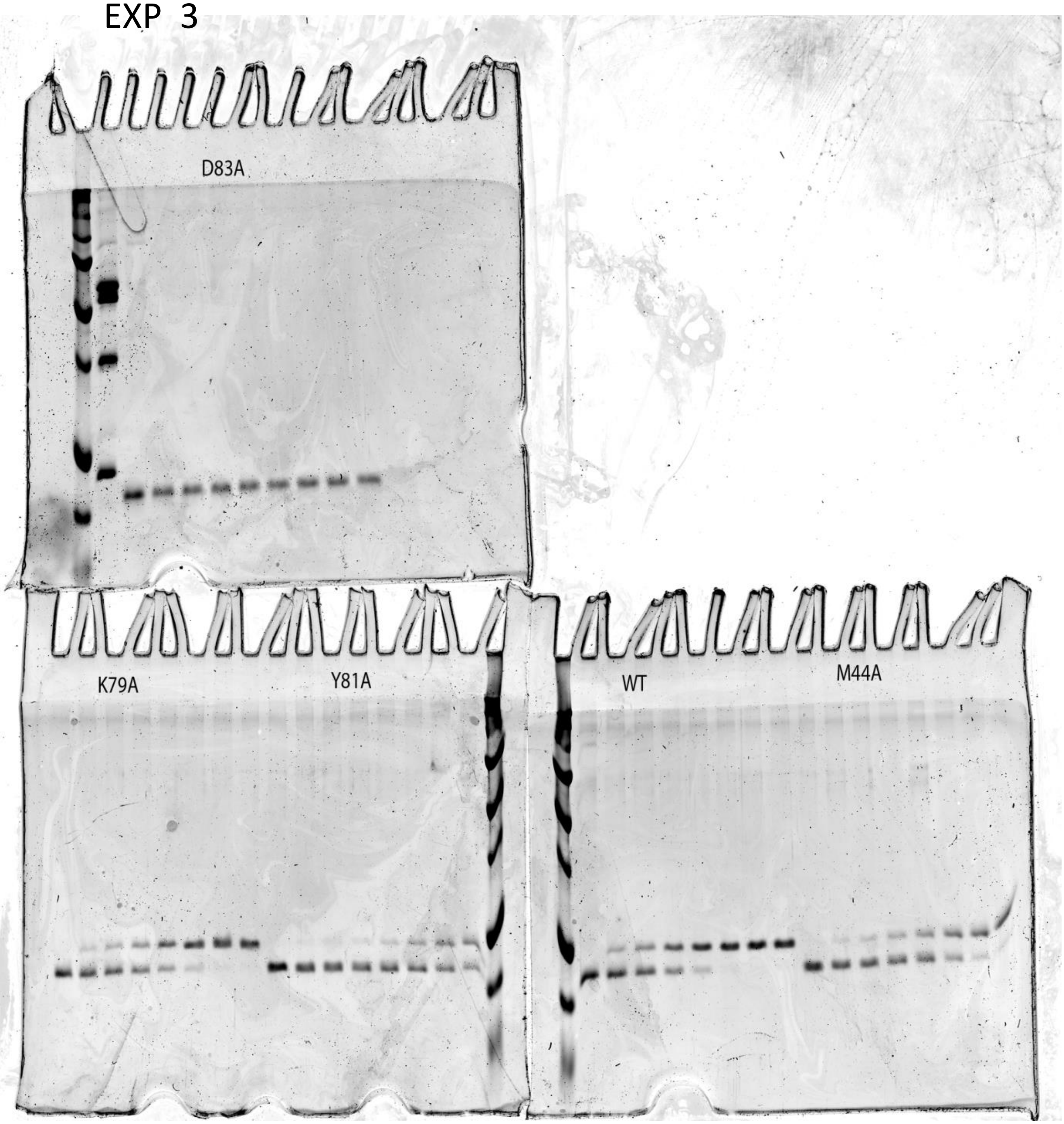

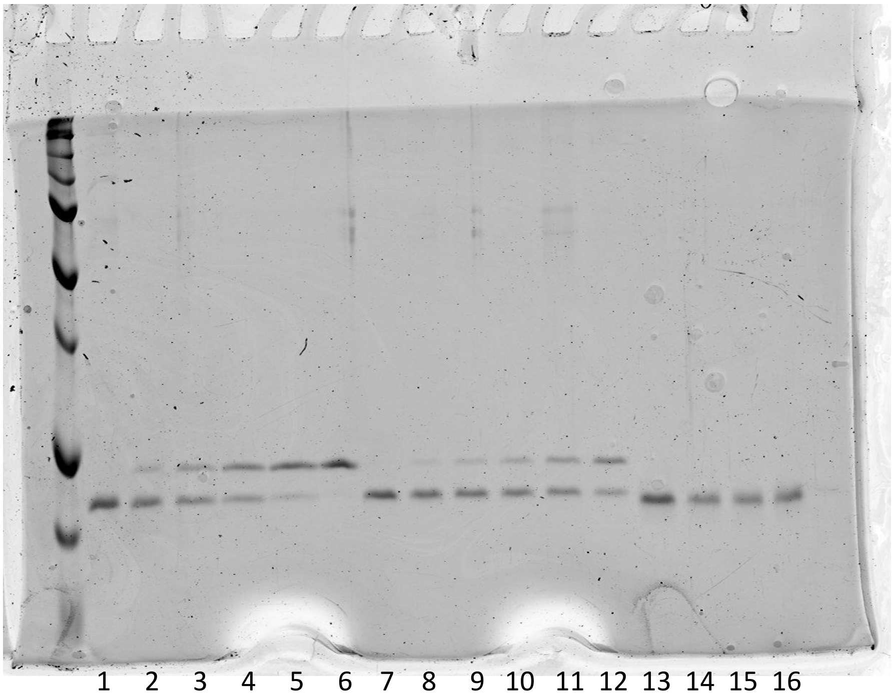

Lane 1-6: WT (Time: 0, 3.5, 6.5, 12.5, 24.5, 48.5min)
Lane 7-12: M44A (Time: 0, 3.5, 6.5, 12.5, 24.5, 48.5min)
Lane 13-16: D83A (Time: 0, 3.5, 6.5, 12.5min)

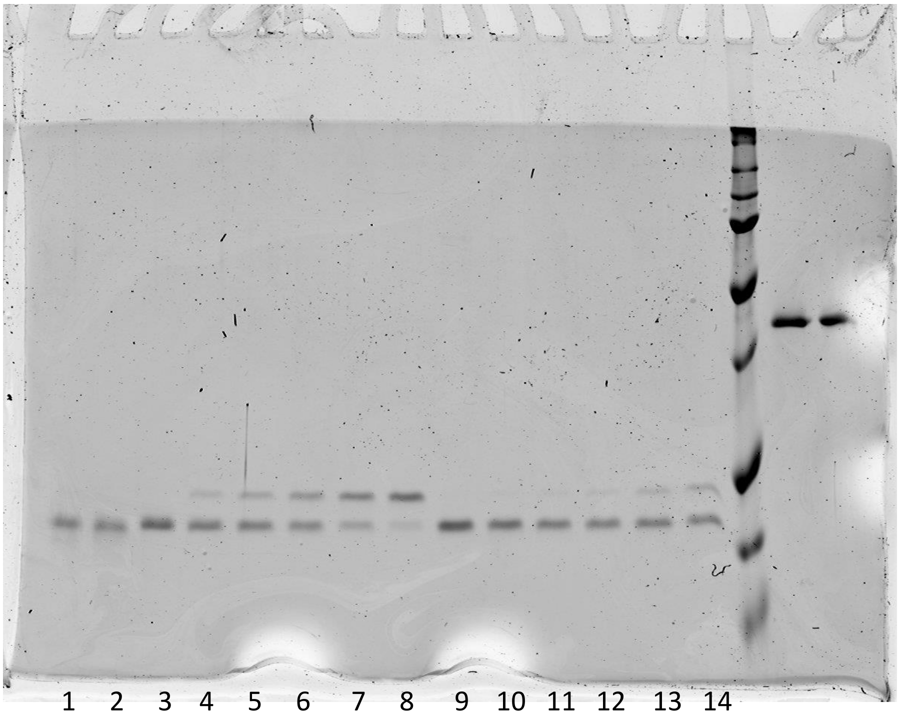

Lane 1-2: D83A (Time: 24.5, 48.5min)
Lane 3-8: K79A (Time: 0, 3.5, 6.5, 12.5, 24.5, 48.5min)
Lane 9-14: Y81A (Time: 0, 3.5, 6.5, 12.5, 24.5, 48.5min)
