# Supplementary material for: Higher-order phosphatase–substrate contacts terminate the integrated stress response
Source: Nat Struct Mol Biol. 2021 Oct 8;28(10):835–46. doi: 10.1038/s41594-021-00666-7 (PMC8500838; doi:10.1038/s41594-021-00666-7)
Supplement: Source Data Extended Data Fig. 2 — Unprocessed gels. [file 41594_2021_666_MOESM13_ESM.pdf]

Original gels for Extended Data Fig. 2

EXP 12.0 Phos-tag gel

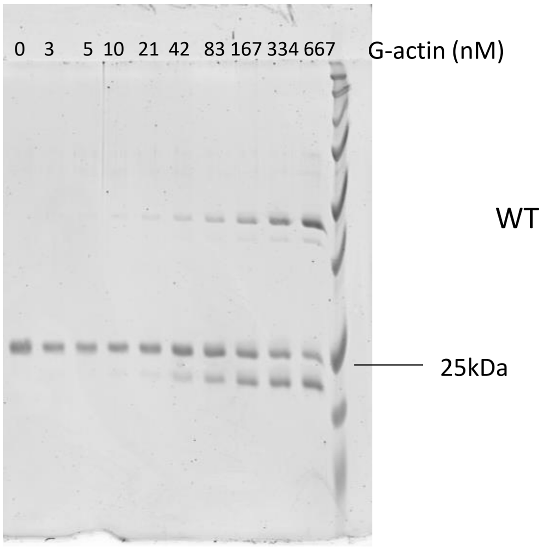

EXP 12.2 Phos-tag gel

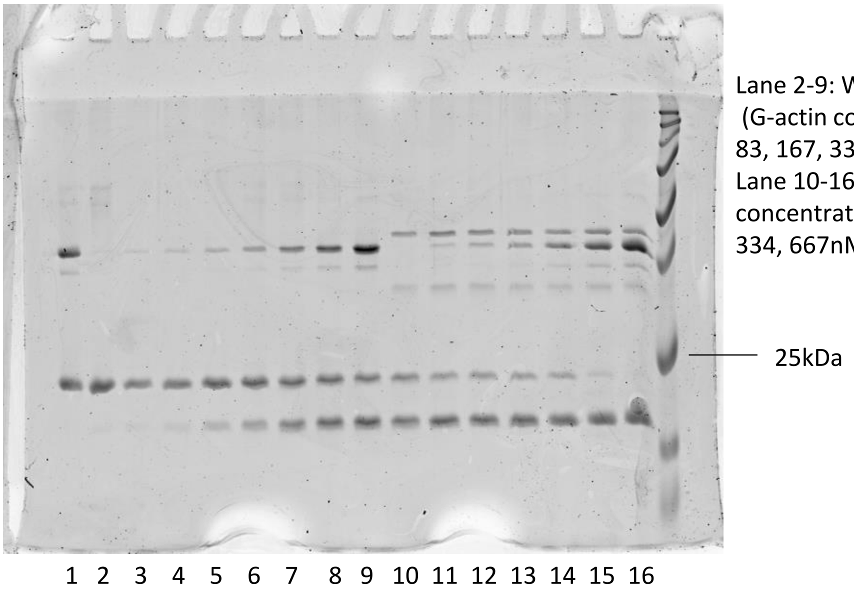

Lane 2-9: WT
(G-actin concentration: 0, 10, 21, 42, 83, 167, 334, 667nM)
Lane 10-16: R595E (G-actin concentration: 0, 21, 42, 83, 167, 334, 667nM)

EXP 12.2 Phos-tag gel

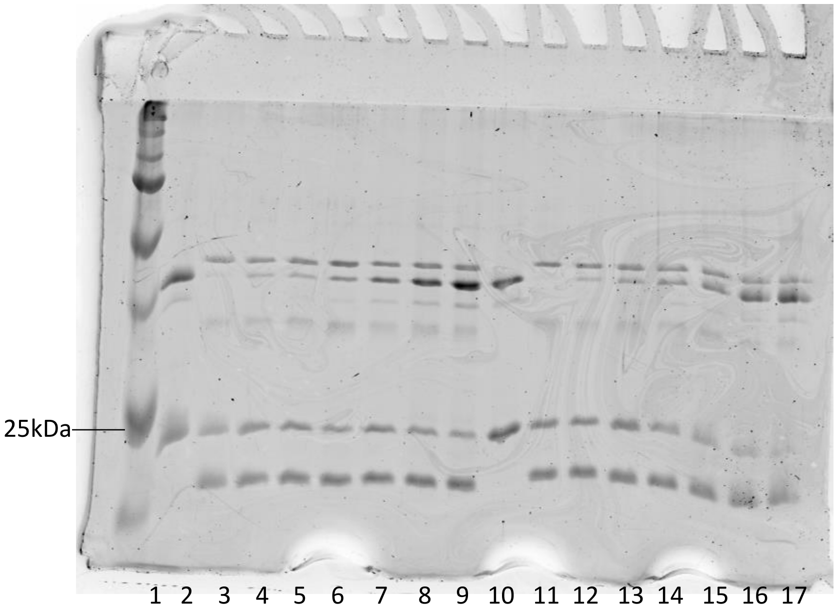

Lane 3-9: F592A_R595E (G-actin concentration: 0, 21, 42, 83, 167, 334, 667nM)

lane 11-17: F592A_R595A(G-actin concentration: 0, 21, 42, 83, 167, 334, 667nM)

EXP 12.1 Phos-tag gel

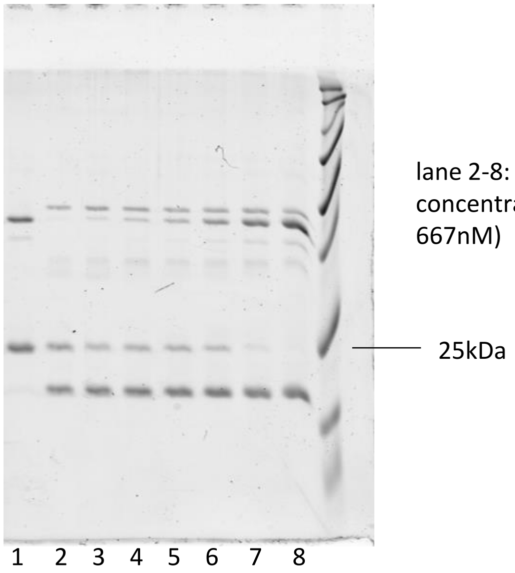

lane 2-8: R595E (G-actin concentration: 0, 21, 42, 83, 167, 334, 667nM)

EXP 12.1 Phos-tag gel

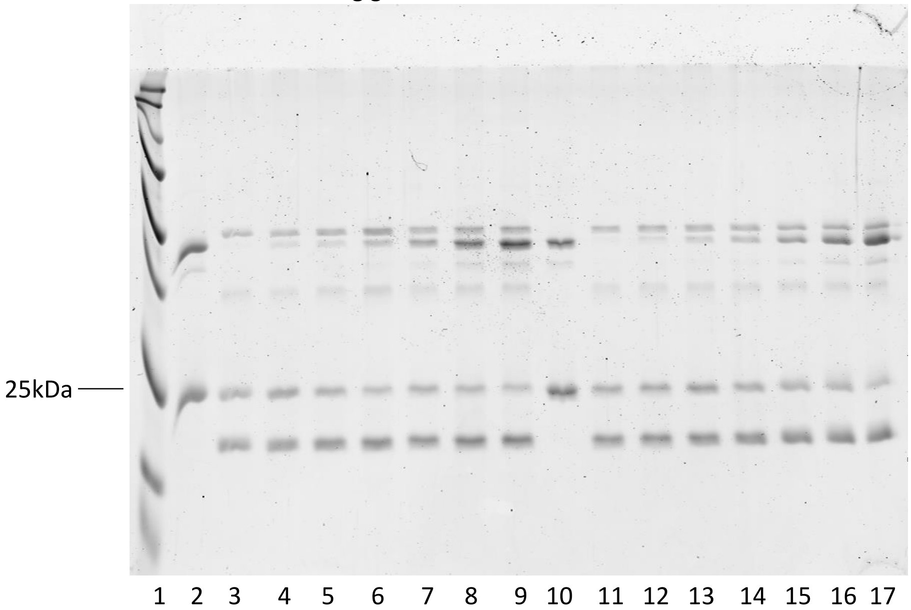

Lane 3-9: F592A_R595E (G-actin concentration: 0, 21, 42, 83, 167, 334, 667nM)

lane 11-17: F592A_R595A (G-actin concentration: 0, 21, 42, 83, 167, 334, 667nM)
